# Where Have All the Interactions Gone? Estimating the Coverage of Two-Hybrid Protein Interaction Maps

**DOI:** 10.1371/journal.pcbi.0030214

**Published:** 2007-11-23

**Authors:** Hailiang Huang, Bruno M Jedynak, Joel S Bader

**Affiliations:** 1 Department of Biomedical Engineering, Johns Hopkins University, Baltimore, Maryland, United States of America; 2 Department of Applied Mathematics and Statistics, Johns Hopkins University, Baltimore, Maryland, United States of America; 3 Laboratoire de Mathématiques Paul Painlevé, USTL, Villeneuve d'Ascq, France; 4 High-Throughput Biology Center, Johns Hopkins School of Medicine, Baltimore, Maryland, United States of America; European Molecular Biology Laboratory, Germany

## Abstract

Yeast two-hybrid screens are an important method for mapping pairwise physical interactions between proteins. The fraction of interactions detected in independent screens can be very small, and an outstanding challenge is to determine the reason for the low overlap. Low overlap can arise from either a high false-discovery rate (interaction sets have low overlap because each set is contaminated by a large number of stochastic false-positive interactions) or a high false-negative rate (interaction sets have low overlap because each misses many true interactions). We extend capture–recapture theory to provide the first unified model for false-positive and false-negative rates for two-hybrid screens. Analysis of yeast, worm, and fly data indicates that 25% to 45% of the reported interactions are likely false positives. Membrane proteins have higher false-discovery rates on average, and signal transduction proteins have lower rates. The overall false-negative rate ranges from 75% for worm to 90% for fly, which arises from a roughly 50% false-negative rate due to statistical undersampling and a 55% to 85% false-negative rate due to proteins that appear to be systematically lost from the assays. Finally, statistical model selection conclusively rejects the Erdös-Rényi network model in favor of the power law model for yeast and the truncated power law for worm and fly degree distributions. Much as genome sequencing coverage estimates were essential for planning the human genome sequencing project, the coverage estimates developed here will be valuable for guiding future proteomic screens. All software and datasets are available in Datasets S1 and S2, Figures S1–S5, and Tables S1−S6, and are also available from our Web site, http://www.baderzone.org.

## Author Summary

The genome sequence of an organism provides a parts list of proteins, but not an instruction manual for assembling the parts into a cell. Assembly instructions now come from experiments such as two-hybrid screens that detect physical interactions between pairs of proteins. Defining the resources required for generating a full interaction map requires accurate estimates of the false-negative and false-positive rates of genome-scale screens. Two-hybrid screens often select a query protein and sample its interaction partners. True partners may be missed, and false partners may be spuriously identified. This sampling process resembles a capture–recapture experiment, except that classical capture–recapture theory assumes no false positives. Novel extensions to capture–recapture theory permit its application to proteomic screens. This new theory provides statistically grounded answers to long-standing questions: false-discovery rates of high-throughput screens (possibly over 50% per unique interaction, but probably no more than 15% per clone); the quality of different screening libraries; protein properties leading to “sticky” or “promiscuous” interactions; the global network topology; and, most importantly, the coverage of existing two-hybrid maps. Models estimate roughly 30,000 total pairwise interactions in yeast and 500,000 to 1,000,000 in metazoans. The majority of these interactions remain to be discovered.

## Introduction

Maps of pairwise protein–protein interactions are being generated in increasing numbers by the two-hybrid method [1]. Genome-scale two-hybrid screens have now been conducted for Saccharomyces cerevisiae (yeast) [2,3], Caenorhabditis elegans (worm) [4], and Drosophila melanogaster (fly) [5]. More recently, screens have been reported for herpesviruses and human [6–8]. These datasets have stimulated large-scale analysis of the topology of protein interaction networks. Limitations in the data, both false positives (spurious interactions reported from high-throughput screens) and false negatives (true interactions missing from the screens), continue to make it difficult to infer network properties [9–11], including distinctions as basic as the difference between Erdös-Rényi (ER), power law [12–14], and other network degree distributions [15].

A recent review points out the challenges in estimating false-positive rates, false-negative rates, and completion to full coverage of protein interaction networks [16]. Virtually every published method falls back to an estimate based on intersections of datasets. For false-positive rates, these methods have large variance when assays have little overlap, and indeed could not be used to analyze the existing large-scale maps for worm and fly. Estimates for false-negative rates based on overlap of datasets may have even larger uncertainty. Finally, global estimates of false-positive and false-negative rates say little about protein-specific properties, including whether certain classes of proteins behave well or badly in two-hybrid screens.

The goal of this work is to develop and apply a statistical model for two-hybrid pairwise interaction screens. Previous methods typically summarize the presence or absence of an interaction as a 1/0 binary variable, and possibly split off a high-confidence core dataset. The method we describe reaches back to the raw counts of observed bait–prey clones. This frees the statistical method from the need for an external gold standard of true-positive and true-negative interactions, or even a second dataset. It permits protein-specific predictions that for the first time permit tests of hypotheses that some classes of proteins are more or less likely to have nonspecific interactions. Finally, estimates of false-negative rates permit statistically grounded confidence intervals for the total number of pairwise interactions present in model organism proteomes.

**Figure 1:**
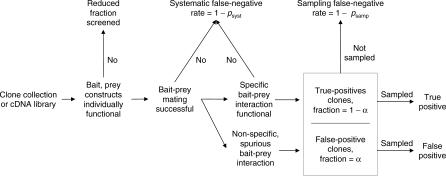
Flowchart for Yeast Two-Hybrid Screens Indicates Systematic and Stochastic Sources of False Negatives and Stochastic Sources of False Positives

A flowchart of a two-hybrid screen orients the discussion by showing where true-positive interaction partners can be lost and where false-positive, spurious interactions may arise (Figure 1). In a two-hybrid assay, one protein is fused to the binding domain (bait construct) of a yeast transcription factor, and a second protein is fused to the activation domain (prey construct). Physical interactions between bait and prey proteins reconstitute transcription factor activity. Due to the expense of the assay, not every protein may be selected to be made into a bait or prey construct. Furthermore, some constructs may not be functional at all due to improper folding or incompatibility with the two-hybrid system. These missing interactions are important to consider when estimating the total number of interactions in a proteome.

High-throughput two-hybrid screens have used multiplexed pairwise tests, either by testing a single bait versus a pool of preys [4,5], or by pooling both baits and preys [3]. Unnormalized prey pools can be generated from mRNA extracted from growing cells. With access to clone collections, pools can be normalized by designing baits and preys individually for each protein or protein domain, then mixing preys in equal proportion. The yeast screen considered here [3] tested 62 normalized bait pools versus 62 normalized prey pools, each pool having approximately 96 genes. The fly screen and worm screen each tested one bait in turn versus both normalized and unnormalized pools.

The testing occurs by using mating or transformation to express both the bait and prey construct in a single yeast cell. True-positive interactions drive reporter genes that permit the yeast cell to grow in selective media. Yeast cells whose bait–prey constructs do not interact are expected to drop out during the population expansion. True positives may also be lost during the population expansion for at least two reasons. First, the mating or transformation may lack enough cells to ensure that every combination is tested. Second, a particular construct may have domain-specific misfolding, making it functional for some interactions but nonfunctional for others.

True interactions that are not represented in the cells following the population expansion are systematic false negatives for a particular screen. False negatives due to insufficient mating/transformation and due to nonfunctional domains could in principle be discriminated by repeating the mating or transformation step and the selective population expansion. Without this additional step, however, losses during the population expansion combine to yield a systematic false-negative rate termed 1 − *p*_syst_, with *p*_syst_ representing the true-positive rate for an interacting pair to survive the population expansion.

Some cells expressing noninteracting proteins may also survive the population expansion, and the final population of cells will be a mixture of true positives and false positives. In Figure 1, the mass fraction of true-positive cells is 1 − *α*, and of false-positive cells is *α*. The ratio of false positives to the total number of true negatives is the false-positive rate. Usually, however, the ratio is with respect to the total number of observed interactions (Equation 31), defined as the false-discovery rate and synonymous with the parameter α.

An ongoing point of contention in two-hybrid screens is the possibility that two proteins that never interact in vivo in the host organism might have a strong, reproducible interaction in vitro in the engineered two-hybrid system. Conversely, proteins with a strong two-hybrid interaction might nevertheless fail to interact in vivo. For the purposes of this work, we assume that such cases are rare and we classify any pair of proteins with a reproducible two-hybrid interaction as a true positive. While the total false-positive fraction *α* may be large, it represents a sum over many different false-positive pairs. Most models, including ours, assume that any particular false positive is rare, with vanishing probability of observing a specific false-positive interaction more than once.

Interactions detected in pooled screens often require sequencing to identify the interacting partners, although advanced pooling designs may improve deconvolution efficiency [17]. Cost constraints limit the number of interactions that can be sampled for sequencing. If the number of clones selected for sequencing is smaller than the number of true interaction partners of a bait, some true partners will certainly be lost. Limited sampling depth also truncates the observed degree distribution for baits. The false-negative rate due to undersampling is termed 1 − *p*_samp_ in Figure 1.

False-discovery rates have typically been estimated by comparing datasets [18–20], suggesting up to 50% false positives, but these analyses can confound false-positive and false-negative error sources. Estimated error rates have large uncertainty because few interactions are observed in multiple datasets. For example, comparing the Uetz and Ito two-hybrid datasets for yeast reveals only 9.1% of the total interactions in common [3], and comparing the two-hybrid interactions with mass spectrometry interactions reveals only 0.6% in common [20]. Similarly, comparison of two fly screens reveals few interactions in common [5,21]. Cross-species comparisons have also revealed little overlap in the reported interactions [4], although protein and network evolution are additional confounding factors.

Efforts to estimate the true number of interaction partners of a protein have used contingency tables for observing an interaction in multiple screens. These methods require that all the interactions be true positives, for example by excluding singleton observations [22], which can reduce the estimated interaction count. A notable exception is previous work in the context of mass spectrometry of protein complexes [23], which used a Bayesian model to infer global parameters for screen-specific false-positive and false-negative rates. These parameters then provided posterior estimates for the probability of a true interaction given results of one or more screens. This work is important in using the number of trials and successes, rather than a single summary yes/no observation, in its probability model; it serves as motivation for developing similar models for the more complicated two-hybrid sampling process involving strong protein-specific effects.

**Figure 2:**
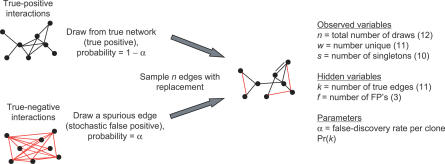
Simplified Schematic Shows the Two-Hybrid Sampling Process In this picture, true-positive interactions (black edges) are sampled uniformly with total probability 1 − *α*, and false-positive interactions (red edges) are sampled stochastically with total probability 1 − α. Sampling is with replacement, and multiple edges between a pair of vertices represent multiple observations of the same interaction. The example shows *n* = 12 edges sampled in the entire network, with *w* = 11 unique edges and *s* = 10 edges that are singletons observed once. The total number of true-positive edges, *k*, and the number of false-positive edges within the sample, *f*, are hidden. The actual experimental data is more complicated, with individual values reported for *n*, *w*, and *s* for each protein used as a bait. The statistical method presented here provides estimates for *k* and *f* together with parameter estimates for *α* and the distribution Pr(*k*).

Quantitative predictions of the amount of work required to identify some fraction of true interactions would be analogous to formulas for genome sequencing [24] and would be useful for planning new experiments [25]. The new work presented here uses the raw screening data to estimate the false-negative rate from undersampling, together with the false-positive rate. A schematic illustrates the sampling process (Figure 2). Interactions are sampled with replacement from two sets, one representing true positives and the other true negatives. The observations are the number of times that each interaction is sampled, which we summarize with three variables: *n*, the total number of samples drawn; *w*, the number of unique interactions within the *n* samples; and *s*, the number of interactions observed exactly once. From these observations we are to estimate the unknown (hidden) values of *k*, the total number of true interaction partners, and *f*, the number of false positives within the sample *n*. We also estimate the parameter *α* representing the fraction of false positives in the mixture (the false-discovery rate), as well as parameters representing the probability distribution for *k*. For simplicity, the illustration suggests sampling interactions in the entire network; in reality, this sampling process occurs separately for each bait, and the estimation of *k* and *f* is performed separately for each bait.

This estimation problem is akin to estimating population sizes or species counts from capture–recapture experiments, estimating vocabulary size from word counts, estimating the number of distinct alleles at a particular locus, and estimating the number of facts in the scientific literature [26–33]. Classic capture–recapture theory permits heterogeneous capturability rates, here analogous to different probabilities of observing each true interaction partner of a bait. The canonical estimator has a simple form: **k̂** = *w* + *s* ^2^/2*k*_2_ [34–36], where *k*_2_ is the number of partners observed exactly twice.

The classic estimator fails in the two-hybrid setting because it does not account for false positives. To our knowledge, false positives have never been discussed in the capture–recapture setting. False positives will vastly inflate the interaction count by adding to the number of singleton observations, *s*, and to the total observed count, *w*. The standard estimator has high variance when the number of observations is small, yielding a small value for the denominator *k*_2_. The estimator fails to converge when each partner is observed only once, yielding *n* = *w* = *s*, *k*_2_ = 0, and **k̂** → ∞.

**Figure 3:**
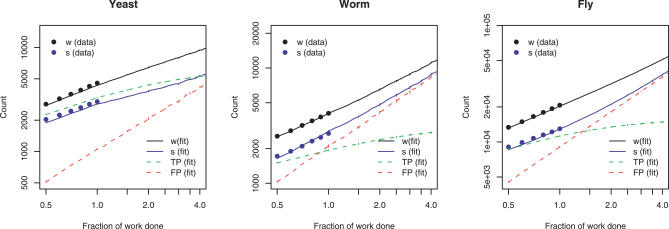
Number of Unique Interactions (*w*) and Singleton Interactions (*s*) Calculated as a Function of the Number of Preys Examined for the Experimental Data (Points) Extrapolations based on half the data are provided for yeast, worm, and fly based on the TPL-MIXTURE model obtained for each.

We present a front-to-back statistical model for both false-positive and false-negative error rates in two-hybrid screens. A glossary of model terms is provided (Table 1). The overall approach is to start by estimating the parameters of a mixture model for true positives and false positives following the population expansion. This permits us to estimate bait-specific false-discovery rates and false-negative rates due to undersampling. We can then back-calculate the false-negative rate due to systematic effects. Putting the results together yields an overall estimate for the false-negative rate of a screen and a basis for comparing interaction lists produced by different efforts. Along the way we examine issues that our model is able to address quantitatively: selecting the best model for the protein degree distribution; correlating false-discovery rates with bait properties such as “sticky” or “promiscuous” domains or hydrophobic regions; and determining the relative performance of prey libraries generated from cDNA libraries or ORFeome collections.

**Table 1:**
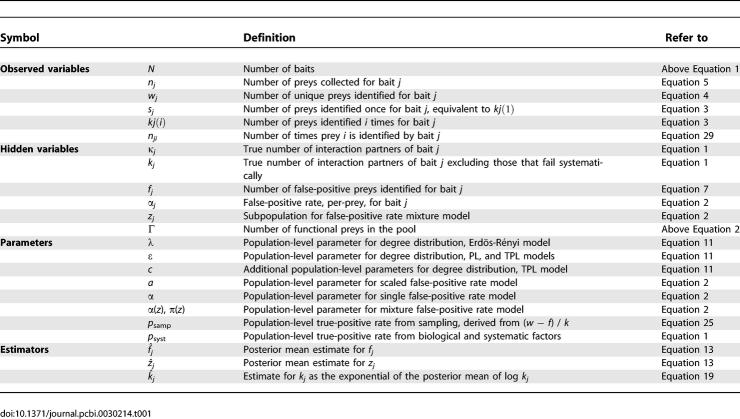
Definitions of Symbols

## Results

### Data Sources

We applied our methods to experimental data from two-hybrid screens conducted in the model organisms yeast, worm, and fly. The key parts of the datasets are the numbers of times that a specific bait identifies each prey, from which all other required values may be calculated. Yeast data was taken from ITO FULL, with clone counts from the IST HIT column [3]. Worm data was from WI5 with clone counts in the NumHitADcDNA and NumHitADORF columns [4]. The worm interactions were from the CORE_1, CORE_2, and NON_CORE sets; interactions annotated as SCAFFOLD (previous screens by the same group), LITERATURE (interactions reported in the scientific literature), and INTEROLOG (interactions inferred cross-species) were excluded. Fly data was from the CuraGen screen with clone counts in the baitprey and preybait columns [5]. A summary of the data sources is provided (Table 2), and a compendium of the data sources is available (Dataset S1).

**Table 2:**
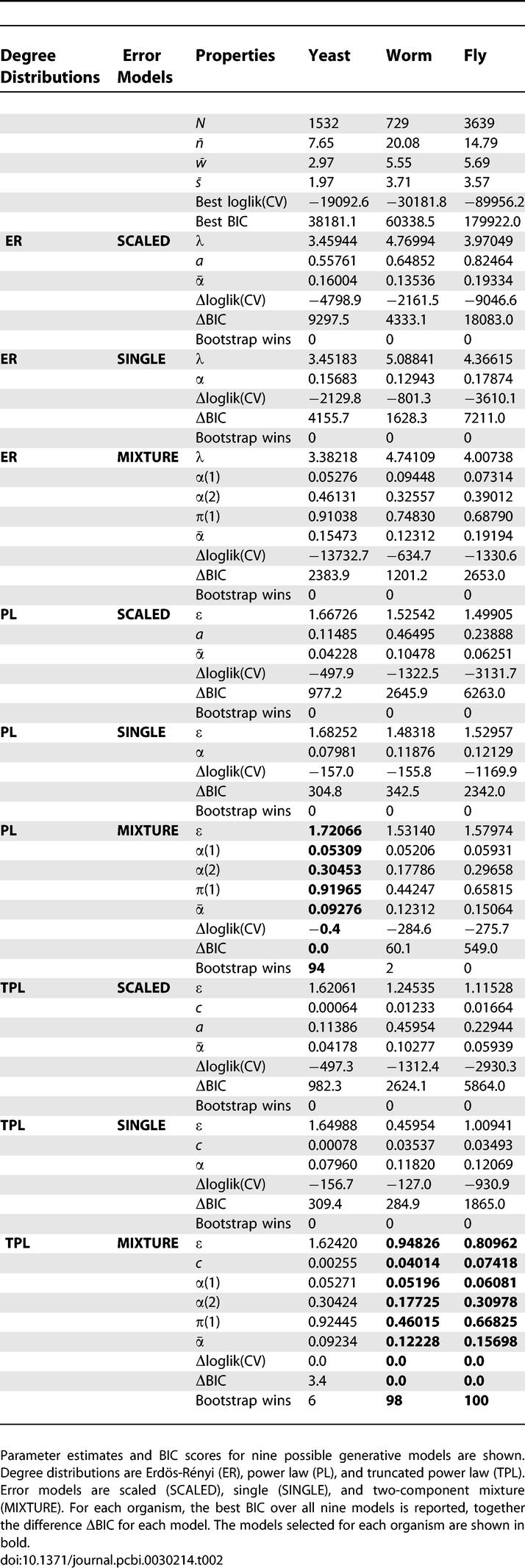
Known Properties of the Experimental Datasets Are Total Number of Baits, *N*; Mean Number of Preys Sampled per Bait, **n̄**; Mean Number of Unique Preys, **w̄**; and Mean Number of Singleton Preys, **s̄**

In collecting these datasets, we noted that many two-hybrid screening publications do not report the clone counts that are required for capture–recapture analysis. This includes one of the two major yeast high-throughput screens [2], a screen for Helicobacter pylori interactions [37], and important recent screens for human protein–protein interactions [6,7]. Part of the motivation of this work is to demonstrate the value of making this type of raw data available for analysis.

### Model Definitions and Assumptions

The relevant variables describing a two-hybrid screen are listed in Table 1 and summarized here. Each of *N* baits is screened against a prey library. For bait *i*, *n_i_* clones from a two-hybrid screen are sampled and the preys are identified. The number of times that prey *j* occurs within bait *i*'s sample is termed *n_ij_*. The number of unique preys within the *n_i_* clones is termed *w_i_*. The number of preys observed exactly once (singletons) is *s_i_*. The *n_i_* clones comprise a mixture of false positives and true positives, but it is not known a priori which are the false positives, or even the total number *f_i_* of false positives.

The goal of our analysis is to estimate the number of false positives, *f_i_*, and the number of true positives that were left unsampled for each bait. Our statistical model makes the following assumptions.

Prey constructs are either functional (with probability *p*_syst_) or systematically lost (with probability 1 − *p*_syst_) with respect to a particular true interaction partner bait construct. Due to possible differences in binding sites, a prey may be functional for one bait and nonfunctional for a different bait. The total number of true positives for a particular bait *i* is termed *κ_i_*, of which *k_i_* ≡ *p*_syst_ *κ_i_* are functional. The parameter *p*_syst_ is estimated from the observed probabilities of bidirectional interactions.

Prey libraries are normalized, with each prey present at equal concentration. True-positive interaction partners are sampled with equal probability with replacement from the *k_i_* functional preys.

False-positive preys occur stochastically, not systematically, with a low probability per prey and negligible probability that any single true negative is sampled twice for a given prey. Thus, clones observed once are a mixture of false positives and true positives; clones observed two or more times are assumed to be true positives.

The cumulative probability that a particular clone is a false positive may be large because it sums over all the possible true negatives. This cumulative false-positive rate is the false-discovery rate per clone, termed *α_i_* for bait *i*, and may be different from bait to bait.

These assumptions are justified in the Materials and Methods section. Even if restrictive, they still provide a necessary starting point for building more complicated models. Given these assumptions, we show in Materials and Methods how false-discovery rates and corrected counts of interaction partners can be determined for each bait.

The posterior estimates for false-discovery rates and interaction counts depend on the functional forms selected for the bait-to-bait heterogeneity in the false-positive rate and the protein interaction degree distribution. We used a variety of model selection criteria, also described in Materials and Methods, that had perfect performance on simulated data.

### False-Discovery Rates

While false positives are a recognized byproduct of two-hybrid screens, there has been little work to investigate bait-to-bait variation in the false-discovery rate. We investigated three models for bait-specific false-discovery rates, described in words here and mathematically in Materials and Methods, Equation 2. The false-positive rate in the model is expressed per sampled clone, rather than per prey in the library (which would be a much smaller error rate) or per unique interaction (which would be a larger error rate).

#### SINGLE error rate model.

The SINGLE error rate model is essentially a null model in which each bait is assumed to have the same error rate determined by a single parameter *α* that is optimized over all the baits used in a screen.

#### SCALED error rate model.

The SCALED model assumes mass balance between true positives and true negatives. True positives are assumed to grow faster. If a protein has many true interaction partners, these colonies will outgrow the true negatives, leading to a smaller error rate. Conversely, if a protein has few or no interaction partners, true negatives will dominate the sampled clones. The false-discovery rate for a protein with *k* interaction partners in this model is *a* / (*k* + *a*), where the parameter *a* is optimized over all the baits used in a screen. The SCALED model predicts that protein interaction degree is negatively correlated with false-discovery rate.

#### MIXTURE error rate model.

The MIXTURE model assumes that baits fall into different error rate classes, with some having higher false-discovery rates than others. There is no a priori assumption correlating error rate with any observation; instead, the class assignments are predicted along with the error rates for each class. In practice, we investigated a two-class model with “good” or low-error baits and “bad” or “promiscuous” high-error baits. This model has three parameters: the class probabilities, and then a single error rate for proteins in each class.

The MIXTURE model outperformed the SINGLE or SCALED models for all organisms (Table 2). The yeast baits were roughly 90% good, with a 5% error rate per sampled clone, and 10% bad, with a 30% error rate. The overall error rate for yeast was 9%. Note that this error rate is per sampled clone. The error rate per unique interaction is 24%, and per singleton interaction is 36% (Table 3).

**Table 3:**
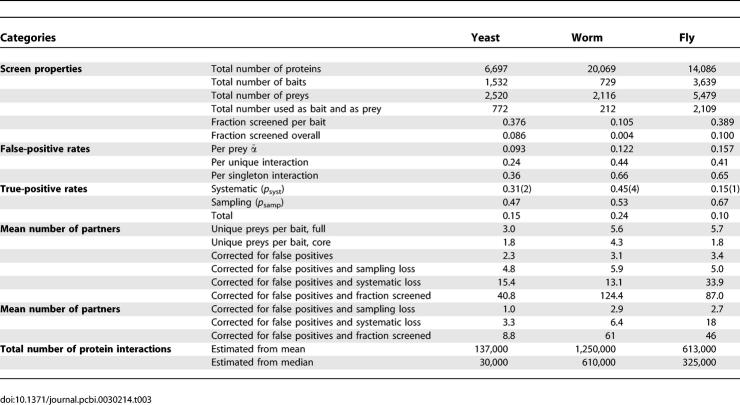
Error Rates and Projections for Full Coverage Provided for Yeast (PL-MIXTURE), Worm (TPL-MIXTURE), and Fly (TPL-MIXTURE) Models

The worm and fly baits showed a more even split between good and bad, with 46% of the worm baits and 67% of the fly baits in the good category. This may reflect improvements in methods for generating bait constructs. The error rates for good baits were 5% for worm and 6% for fly; the error rates for bad baits were 18% and 31%. The overall false-discovery rates were 12% and 16% per sampled clone in worm and fly, corresponding to error rates of about 40% per unique interaction and 65% per singleton interaction.

These error rates are in general agreement with estimates obtained by comparing datasets (Introduction). Because our results are bait-specific, however, we can test popular hypotheses for the sources of false positives in two-hybrid screens. Suggestions have included that certain domains are likely to participate in nonspecific interactions, or more generally that hydrophobic interactions can generate power law degree distributions entirely due to spurious nonspecific interactions [38].

For parametric tests, we used the ratio of the posterior estimate of the number of false positives for bait *i*, **f̂*_i_*, to the total number of clones sampled, *n_i_*, as a posterior estimate α̂*_i_* for the false-discovery rate per sampled clone. Test statistics for specific classes of proteins summed the individual (*n_i_*, **f̂*_i_*) values for proteins within the class, then used the ratio of the sums as the class estimate.

#### Prey library quality.

Both the worm screen and the fly screen used two distinct prey libraries: one library was generated from a sequence-verified ORF collection, and the second from a cDNA collection. An important motivation for using an ORF collection is that near-perfect normalization of prey concentrations and higher-quality prey sequences will reduce the error rate of a screen. We were able to test that hypothesis through analysis of the worm data. Unfortunately, the fly data did not include sufficient detail to permit a similar test.

We again found strong evidence for the mixture model for false-discovery rates. With the ORF library, the overall good class probability was estimated as 90%, while with cDNA, only 46% were classified as good. The error rates for the good category were 8% in each case. The error rates for the bad category were 80% for the ORF library and 33% for the cDNA library. The overall error rate was lower for the ORF part of the screen, 16% versus 21%.

For a more quantitative comparison, we examined a model in which the posterior estimate of the error rate for a bait in the cDNA screen depends linearly on the error in the ORF screen. This model yielded a *p*-value of 0.0005 with a slope of 0.34 (95% confidence interval [0.15, 0.53]), demonstrating that the relative error rate of a bait is consistent from screen to screen. A rough estimate for the bait-specific increase in error for the cDNA library relative to the ORF library can be obtained by forcing the intercept in the linear model to be zero, which yields a slope of 1.34 (*p*-value < 2 × 10^−16^). Thus, we conclude that each bait has a false-discovery rate that is approximately 34% higher in the cDNA screen than the ORF screen.

#### Promiscuous and chaste domains.

We tested the hypothesis that certain protein domains are more likely to yield false-positive interactions. PFAM assignments were used to characterize protein families and domains [39]. For each domain, we calculated one-sided *p*-values for both higher and lower numbers of false positives estimated than expected by chance. The *p*-values were then corrected for multiple testing by multiplying by twice the number of domains tested in each organism. Several domains were identified as promiscuous, having significantly higher false-discovery rates than average (Table 4).

**Table 4:**
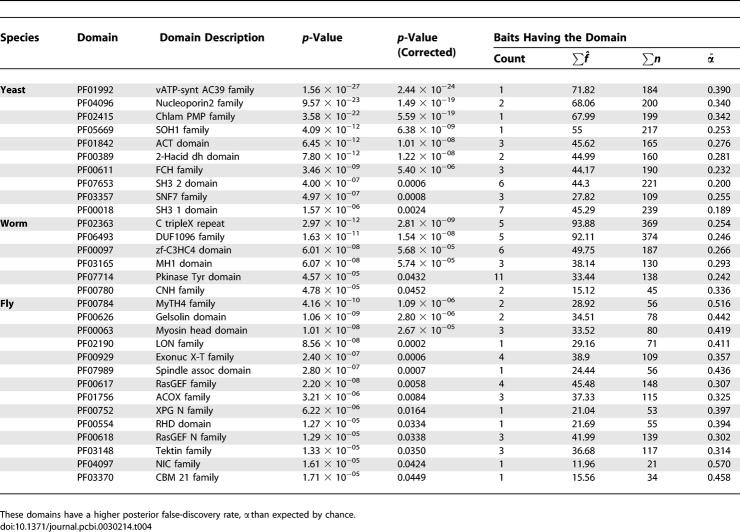
Promiscuous Domains

A major theme is the appearance of domains corresponding to membrane-bound proteins (vacuolar ATP synthase, Chlamidia PMP, nucleoporin, and NIC nuclear pore transport). Other domains occur in tyrosine kinase and other signaling pathways (SH3, RasGEF). Note, however, that not all signaling kinases have high false-discovery rates. Indeed, protein kinases considered as a group actually have significantly lower false-discovery rates than average. Thus, we reject a possible explanation that the absence of scaffold proteins that provide specificity in protein kinase signaling [40] leads to a high false-positive rate. Instead, we suggest that it is particularly the membrane-bound signaling proteins that have high false-discovery rates, consistent with high false-discovery rates observed for other protein domains with membrane localization. This hypothesis is further tested using cellular compartment annotations (see the section Gene Annotations).

Domains involved in transcription are also represented as having high false-discovery rates. It is possible that these proteins have a low level of auto-activation leading to spurious false positives. Finally, general cytoskeleton and protein binding domains occur in the list.

Only a few domains have significantly reduced false-discovery rates (Table 5). These domains include ribosome and ribonucleoprotein biogenesis and DNA binding activity. It is possible that the DNA binding activity is more specific in these domains, for example limited to single-stranded DNA (Translin family) or specific sequences (BESS motif) as compared to the high false-discovery rate proteins.

**Table 5:**
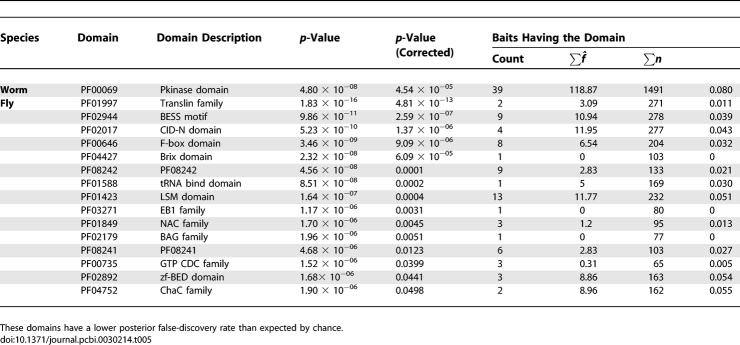
Chaste Domains

We also tested the hypothesis that domains that are prevalent in an organism may yield false positives by spurious weak cross-reactivity with the binding partners of other proteins within the same family. This hypothesis was tested using a linear model in which the overall posterior false-discovery rate for the proteins within a protein family depends on the family size. The two-sided *p*-values for yeast, worm, and fly were 0.38, 0.97, and 0.02. Despite the significant *p*-value for fly, the *R* ^2^ for the fit was only 0.004. Thus, if this effect exists at all, it is a minor contributor to the false-positive rate.

#### Gene annotations.

We performed a similar analysis for significantly high or low false-discovery rates for bait proteins based on their Gene Ontology annotations [41]. Analysis proceeded according to the three major ontologies: cellular component (Table S3), biological process (Table S4), and molecular function (Table S5).

All three ontologies provide evidence that membrane proteins have higher false-discovery rates in all three species. Cellular component categories with elevated false-discovery rates include the Golgi membrane (evidence from fly), the nuclear membrane and nuclear pore (yeast), the vacuolar membrane (yeast), other organelle membranes (yeast), and membrane-bound organelles (worm). Grouping by biological process, annotations show higher false-discovery rates for processes related to cellular localization, ion homeostasis, pH homeostasis, ion transport, and nuclear transport (yeast), which all involve movement of molecules across biological membranes. Localization (worm and yeast) also shows a high false-discovery rate. Molecular functions involving membrane transport, such as ATPase-coupled transport and general transporter activity (yeast), also show elevated false-discovery rates.

While ion transport proteins have high false-discovery rates, ion-binding proteins do not. Cation binding proteins (yeast) have significantly lower false-discovery rates than average, as do proteins with molecular functions of phospholipid binding, nucleic acid binding, and protein binding (fly).

A similar distinction appears to be in effect for proteins with enzymatic function. Enzymes that participate in signaling pathways have lower false-discovery rates; enzymes that participate in basic biosynthesis have higher false-discovery rates. This hypothesis is supported by results from the molecular function analysis. Transferases, including methyltransferases (fly) and kinases (worm), have significantly lower false-discovery rates than average. These biochemical reactions are typically important for signaling. Consistent with this result, proteins with biological processes related to signal transduction (worm), neurogenesis and neuron morphogenesis (fly), cell part morphogenesis (fly), laval behavior (fly), and memory (fly) have significantly lower false-discovery rates than average.

Enzymatic functions with higher false-discovery rates than average include exonuclease and metalloendopeptidase activity (fly) and oxidoreductase activity (yeast). Cofactor binding proteins (yeast), which often participate in enzymatic reactions, also have elevated false-discovery rates. Biological processes showing higher false-discovery rates include cofactor biosynthesis (yeast), nucleotide biosynthesis (yeast), and mRNA metabolic processes (yeast).

#### Hydrophobic interactions and protein length.

The above results indicating a higher false-discovery rate for membrane proteins suggest two possible routes toward nonspecific interactions: (1) Membrane proteins have hydrophobic residues that associate nonspecifically. Indeed, statistical models have suggested that nonspecific hydrophobic interactions are responsible for power law degree distributions observed in two-hybrid screens [38]. (2) Apart from hydrophobicity, membrane proteins may become disordered in the nuclear environment, leading to nonspecific interactions.

These hypotheses can be assessed by testing for significant association between hydrophobicity and false-discovery rates. Several accepted hydrophobicity scales are available, including those due to Kyte-Doolittle [42], Eisenberg [43], Cornette [44], and Rose [45]. For each hydrophobicity scale, we summed the values for each residue of a bait protein to obtain a single summary score for the entire protein chain. We then tested the significance of a model in which the posterior estimate for the false-discovery rate of a bait, defined as **f̂*_i_*/*n_i_* for bait *i*, depends on its hydrophobicity (Table 6).

**Table 6:**
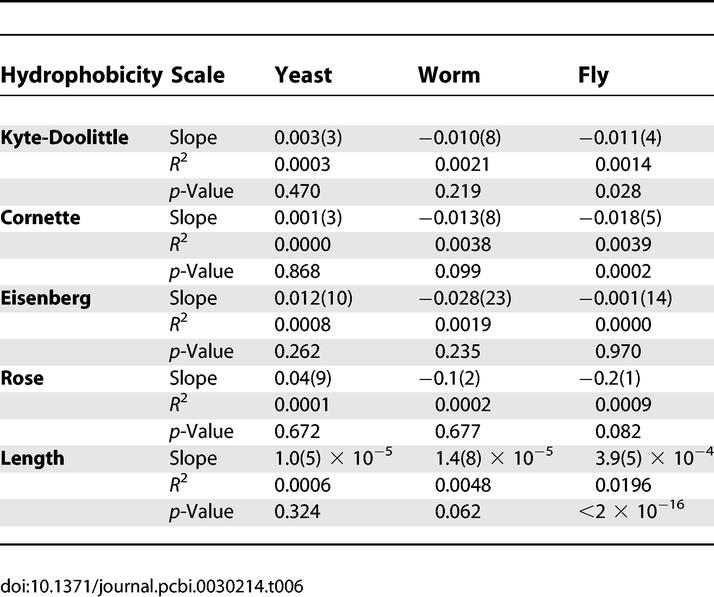
Correlation of False-Discovery Rates with Hydrophobicity Scales and Length

The results of this analysis fail to show a conclusive relationship between hydrophobicity and false-discovery rates. The results of the fly screen provide some support for the hydrophobicity hypothesis, with *p*-values of 0.03 and 0.0002 according to the Kyte-Doolittle and Cornette scales. Nevertheless, the *R* ^2^ values are negligible, 0.001 to 0.004, indicating that any effect is very very small. The *p*-value from the Rose scale is 0.08, not significant according to a two-sided test but significant for a one-sided test. The *p*-value for the Eisenberg scale is not statistically significant for fly. Furthermore, none of the hydrophobicity scales yields a significant model for either yeast or worm.

A possible source of error in the hydrophobicity analysis is that the hydrophobicity of the entire protein is used for the linear model. The effect of hydrophobic patches may be masked by the variance of the sequence as a whole. We expect, however, that any conserved hydrophobic domains are included in PFAM.

We performed a similar analysis based on protein length. No significant correlation was observed between length and false-discovery rate for the baits in the yeast and worm screen. A highly significant correlation was observed in the fly screen. Again, though, the *R* ^2^ value of 0.02 indicates that any effect is negligible.

In summary, while membrane proteins have higher false-discovery rates, this effect may be due to disordering of protein structure in the nucleus rather than to pure hydrophobic interactions between properly folded proteins.

#### Protein degree.

We finally investigated whether protein degree correlates with false-discovery rate by testing linear models for dependence of the posterior error rate, α̂*_i_*, on the number of estimated true-positive interaction partners observed, *w_i_* − **f̂*_i_*, and the estimated protein degree, **k̂*_i_*, described in the following section. We also investigated the number of clones sampled *n_i_*, an experimental parameter. Due to the large range of interaction counts, the analysis used the log-transforms log(*w* − **f̂**), log(**k̂**), and log(*n*) (Table 7).

**Table 7:**
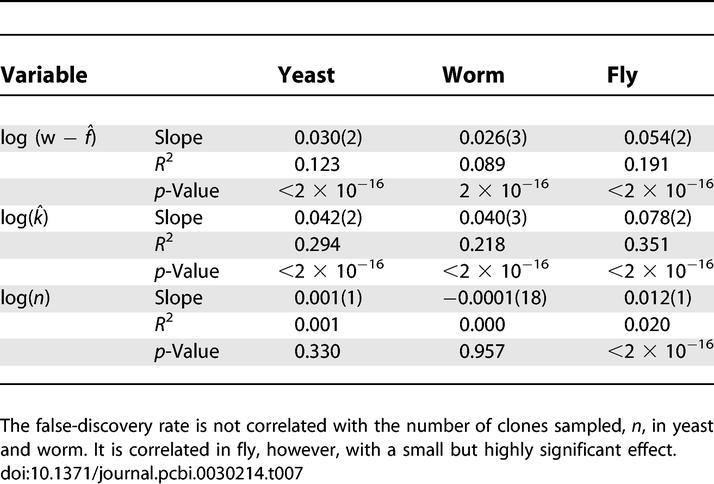
The False-Discovery Rate for a Bait Protein, **f̂**/*n*, Positively Correlated with the Estimated Number of True Interaction Partners That Are Observed, w − **f̂**, and the Total Number, **k̂**

The false-discovery rate shows a strong dependence on estimates of both the observed and total true-positive interactions for a protein, with *R* ^2^ values in the range 0.1 to 0.4. This correlation arises because proteins with a large number of true interactions and proteins with a high false-discovery rate will both yield many singleton interaction partners. While the statistical model attempts to discriminate between these two sources of singleton interactions, some correlation remains.

The number of clones sampled per bait is often determined in advance of conducting a screen, and may not vary much from bait to bait. Under these conditions, the false-discovery rate is anticipated to be independent of *n*. This is indeed the case for yeast and worm. For fly, a very small but significant positive correlation is seen, with *R* ^2^ = 0.02. In the fly screen, some baits yielding new interaction partners were indeed sampled deeper (personal communication, L. Giot). Even in the fly screen, however, baits with the most preys sampled are not necessarily the baits with the greatest number of interaction partners. The most heavily sampled bait was CG5063, with 233 preys, one observed 232 times and the other observed once. And of the ten most heavily sampled baits, six have predicted degree lower than the average predicted degree.

#### False-discovery rate summary.

To summarize, our analysis strongly supports a heterogeneous false-discovery rate among bait proteins and provides a rigorous basis for identifying factors that contribute. High-quality bait and prey libraries from ORFeome-type collections are shown to reduce false-positive rates by one-third.

An important biological theme that correlates with high false-discovery rates is membrane localization. This correlation is observed based on protein domains structure and cellular compartment annotations. Membrane localization appears to be more relevant than a broader categorization based purely on protein hydrophobicity. A second theme is that proteins with enzymatic activity appear to have lower false-discovery rates when the activity is related to signaling pathways, and higher false-discovery rates if the activity is related to biosynthetic pathways.

The overall estimates for false-positive rates, per unique interaction, are roughly 25% for yeast and 40% to 45% for worm and fly. Previous estimates for this yeast dataset range from 70% to 90%; estimates for worm and fly have been considered untrustworthy due to limited data (see [16]).

The source of the difference may hinge on the interpretation of bait proteins that identify several singleton preys. These baits are either hub proteins with many true interaction partners, or proteins with high false-discovery rates and nonspecific interactions. Previous methods attempt to perform this classification by cross-comparing with gold-standard interactions. Our method performs this classification by examining the histogram of preys identified two times, three times, and so on, then back-calculating the number of preys that should have been observed once.

### Protein Interaction Degree Distribution

We selected three representative functional forms as possible models for the probability that a bait protein has *k* functional interaction partners in the prey library, described in text here and more formally in the section Theory.

#### Erdös-Rényi (ER) or Poisson model.

This model corresponds to the Erdös-Rényi random graph model of a uniform probability of an interaction between any two proteins, which has the limiting form of a Poisson distribution. The single parameter of this model is determined by the mean value of *k*.

#### Power law (PL) model.

The power law (PL) model describes a scale-free distribution in which the probability of a protein having *k* partners is proportional to 1/*k^ɛ^*. The exponent *ɛ* in this one-parameter model is determined by the mean value of log *k*. This type of network arises from network growth algorithms with preferential attachment of new nodes to existing nodes [46], as could be expected to occur from gene duplication events.

#### Truncated power law (TPL) model.

The truncated power law (TPL) model reduces the probability of high-degree proteins by introducing exponential decay as a second parameter. The TPL model includes the PL model as a special case. Truncation is a natural consequence of the finite size of the proteome, and can also arise from a network with modularity.

Each of these protein degree distribution models was tested in conjunction with each of the error models during model selection. The model selection criteria, which included corrections to penalize the TPL model for having more parameters, had perfect performance on data simulated from each of these models.

Of the one-parameter models, PL is clearly superior to ER for the yeast, worm, and fly datasets (Table 2). This finding is important because light sampling of an ER network (and networks with other degree distributions) can yield a bias toward a power law degree distribution [10,47]. Our methods for estimating the true protein degree correct for light sampling, a claim substantiated by perfect model selection for simulated data (Table S2).

Adding an exponential decay parameter to obtain a TPL provides an improved model for the worm and fly data. For the yeast data, however, the truncation does not improve the fit. A possible explanation for a PL yeast network and TPL worm and fly network is that truncation can be due to high-level network partitioning [15]. The truncation in worm and fly may therefore be due to metazoan tissue-level organization, absent in single-celled yeast.

As described in Materials and Methods, the degree distribution parameters permit posterior estimates of **k̂*_i_*, the number of functional interaction partners of bait *i* in the prey library. Our choice for the posterior estimate **k̂*_i_* is exp〈log *k_i_*〉, the exponential of the posterior mean of the logarithm of the degree. This form of the estimator is suggested by the observation that 〈log *k*〉 is a sufficient statistic to determine the single parameter of a power law network, Equation 18, and that the experimental networks are long-tailed even if not purely PL.

The **k̂*_i_* values can then be used to estimate false-negative losses due to two distinct sources: stochastic undersampling of functional preys, with methods described in the section Parameter Estimation; and systematic loss of nonfunctional preys, based on a bidirectional analysis described in Materials and Methods under the section False-Negative Rates.

### False-Negative Rates

#### False negatives due to undersampling.

The stochastic false-negative rate for an entire screen may be estimated as ∑*_i_*[**k̂*_i_* − (*w_i_* − **f̂*_i_*)]/∑*_i_*k̂*_i_*, where the numerator represents the total number of true interactions minus the observed interactions, and the denominator represents the total number of true-positive interactions. Stochastic losses could in principle be corrected by deeper sampling of two-hybrid clones. One minus the stochastic loss rate is termed the sampling true-positive rate and is provided in Table 3 for each of the organisms.

Our results indicate that about half of the interactions that could have been observed were observed in each of the screens: 47% for yeast, 53% for worm, and 67% for fly. To our knowledge, these are the first estimates of stochastic undersampling rates for two-hybrid screens. The roughly 50% true-positive rate for functional clones is remarkably high given the low overlap between screens done in the same organism. The dominant contribution to false negatives may therefore be systematic losses from nonfunctional or absent preys (see the section False Negatives due to Systematic Loss) rather than stochastic undersampling.

Because the estimates for sampling coverage seemed higher than typically assumed for two-hybrid screens, we developed a cross-validation scheme to test these predictions using the experimental data (see the section Cross-Validation with Experimental Data). In short, we used half of each dataset to estimate model parameters, which were then used to predict the number of total interactions and the number of singleton interactions in the remaining half. The predictions from cross-validation perfectly overlay the experimental data for worm and fly, and are in excellent agreement for yeast (Figure 2).

The cross-validation method is also able to predict the number of true-positive and false-positive interactions within the data, and to extrapolate for larger datasets. The extrapolated curves for true positives in Figure 2 indicate that the number of clones sampled could be doubled without seeing decreasing returns of true positives, but could not be increased much beyond that.

Even though half of the functional preys are predicted to be present in the datasets as published, identifying these true interaction partners remains a challenge. The true-positive rates drop to 21%, 31%, and 42% for yeast, worm, and fly if singleton interactions are discounted. While singletons are not typically incorporated into high-quality subsets, they can be very useful as part of data integration methods that combine multiple data sources for greater confidence [20,48–50].

#### False negatives due to systematic losses.

Once the false-negative rates have been corrected bait-by-bait for undersampling, bidirectional analysis can be used to estimate the additional false-negative rates due to systematic losses (see the Materials and Methods section False-Negative Rates). These estimates are built from a subset of data representing true positives that are identified in one direction and which could have been identified in the reverse direction. The calculations are restricted to proteins that have at least one interaction recorded as a bait and a prey to exclude constructs that may be completely nonfunctional. The systematic false-negative rates, denoted 1 − *p*_syst_, are estimated as 0.69 for yeast, 0.55 for worm, and 0.85 for fly (Table 8).

**Table 8:**
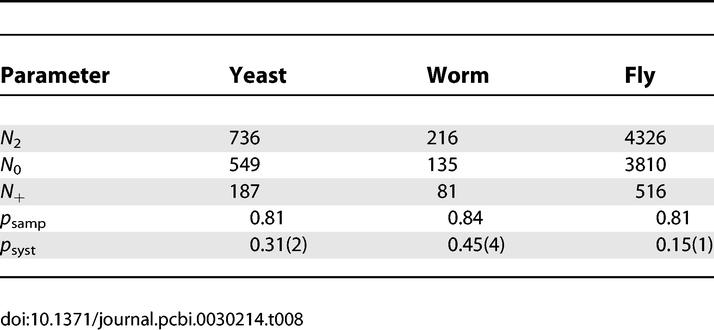
Parameter Estimates for the True-Positive Rates for Avoiding Systematic Losses

This result may indicate a high quality set of bait and prey constructs for worm. These constructs came out of an effort to clone the worm ORFeome [51] and may be of higher quality than the yeast set, which had been generated earlier. The fly false-negative rate may be higher due to greater reliance on cDNA libraries and reduced effort to confirm each construct in the collection set. In particular, a cDNA that is not full-length may lack domains responsible for certain interactions.

#### Overall false-negative rates.

The overall false-negative rate is 1 − *p*_syst_ *p*_samp_. The corresponding true-positive rates, *p*_syst_ *p*_samp_, are provided in Table 3: 15% for yeast, 24% for worm, and 10% for fly. These false-negative rates provide an immediate explanation of the low number of interactions seen in multiple screens: two screens that are each only 10% complete will only share 1% of their interactions, assuming perfect concordance of the baits and preys screened.

High-throughput screens have by design used different strategies for the sampling space—the baits and pairs tested—and the depth of clones sampled within this space [52], which further reduces the intersection in practice.

#### Comparison with previous per-protein estimates.

To our knowledge, there has been only one previous method for estimating the true number of interaction partners of a protein in a two-hybrid screen, based on the number of interactions in the intersection of two independent screens and which we denote *k* ^∩^ [22]. This previous method is limited by requiring that each interaction is a true positive, and thus takes information from only the high-confidence component of an interaction screen. Yeast is the only organism where two large-scale screens have considerable overlap in baits and preys used [2,3]. Predictions using the *k* ^∩^ estimator are possible for only the 631 proteins that were used in both screens. Of these 631 proteins, 307 are predicted by *k* ^∩^ to have a single interaction partner, 140 are predicted to have two interaction partners, and only 34 are predicted to have more than ten interaction partners.

In contrast, the methods presented here are able to make predictions for all 1,532 bait proteins used in the screen. Furthermore, by making use of the full tabulation of clone counts for each prey, rather than just the number of high-confidence preys, the method is able to discriminate between baits that are high-degree due to a high false-positive count and baits that are high-degree due to many true interaction partners.

The entire set of predictions **k̂** from Equation 19 and κ̂ from Equation 31, is compared with the previous estimator *k* ^∩^ in Dataset S2, with degree distributions depicted in Figure S5. Jumps in the degree distribution **k̂** occur due to large classes of baits for which every prey is a singleton (Table S6). Counts of reported interaction partners from other screens are from BioGRID [53]. Representative cases are summarized (Table 9) with selected examples discussed below.

**Table 9:**
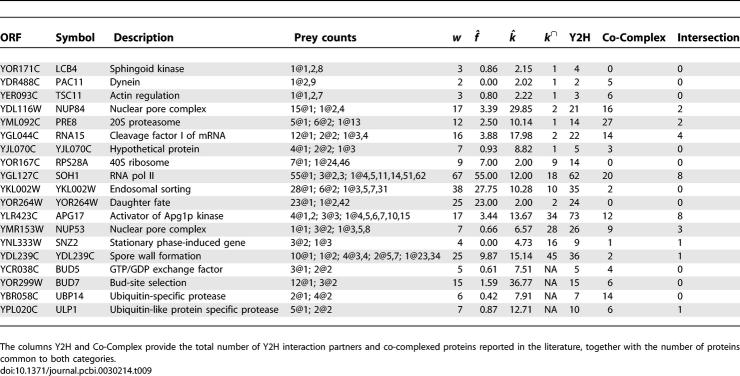
Protein Interaction Count Predictions Provided from This Method, **k̂**, and from a Previous Method, *k* ^∩^

*Agreement on a short, converged list of interaction partners.* For some proteins, the sampling from the two-hybrid screen seems to have converged on an accurate, short list of interaction partners with agreement between **k̂** and *k* ^∩^. One example is the gene TSC11, involved in actin regulation. The corresponding bait had ten clones sampled, in which one interaction partner appeared seven times, a second partner appeared two times, and a third partner appeared one time. The estimate **k̂** predicts 2.22 total partners and gives an 80% probability that the singleton partner is a false positive. The *k* ^∩^ estimator suggests a single interaction partner. This protein has six co-complex members, but there is no overlap between the co-complexed proteins and the two-hybrid interaction partners.

*Disagreement with a new, higher estimated interaction count.* In these examples, the new estimator suggests that several of the singleton observations are true interaction partners, yielding a high interaction count. The *k* ^∩^ estimator does not include the singletons, leading to a low estimate of only one or two interaction partners. An example is the RNA15 gene product, which had 12 singleton preys, two preys observed twice, and one each observed three and four times. From the 16 unique interactors, the *k* ^∩^ method suggests two true positives. Our estimator suggests that eight of the 12 singletons are true positives. After correcting for undersampling, the estimator suggests 18 interactions. This protein has 14 known co-complex members, and four overlap with the two-hybrid data.

*Agreement with many false positives filtered out.* Other baits with a small predicted number of interaction partners by **k̂** and *k* ^∩^ actually have a large raw interaction count and a corresponding prediction of many false positives. Two examples from this category are YKL002W, involved in endosomal sorting, and YOR264W, involved in daughter cell fate. The YKL002W bait had 86 clones sampled, with 28 singleton observations and 38 unique partners. The mixture model predicts that all 28 singletons are false positives, and suggests 10.3 true interaction partners. The *k* ^∩^ method suggests ten interaction partners as well. Only two co-complexed proteins have been detected for this gene, and neither overlaps with the two-hybrid partners. Results for the YOR264W bait are similar. Of 67 clones sampled, 23 were singletons. An additional prey was identified twice, and a third was identified 42 times. Both **k̂** and *k* ^∩^ suggest that the true interaction count is two, as opposed to the raw count of 25 unique partners. No co-complexed proteins have been reported for this protein.

*Disagreement with a new, lower estimated interaction count.* Observing multiple partners multiple times provides strong evidence that sampling has converged, with **k̂** not much different from the raw number of unique partners. The *k* ^∩^ estimator can give a larger estimate in these cases, possibly due to increased variance from a small denominator. An example is the NUP53 gene product, with one singleton, three partners observed twice, and one partner each with three, five, and eight clones. The new estimator gives a 66% chance that the singleton is a false positive and suggests seven interaction partners overall. The *k* ^∩^ estimator suggests 28 interaction partners. This protein has nine reported co-complex members, with three overlapping with the two-hybrid partners.

*New ability to provide an estimate.* The **k̂** estimator provides estimates for baits that have only been run in a single screen. For example, the BUD5 GTP/GDP exchange factor has three singletons, and two partners observed twice each. These results suggest that sampling may be close to converged, with roughly eight partners expected. This protein has four co-complex members, although none overlaps with the two-hybrid partners.

#### Comparison with previous false-negative rate estimates.

Global false-negative rates have been estimated in the past by comparing a high-throughput interaction set to a gold-standard set extracted from the literature. We have carried out this analysis using curated interactions from the Database of Interacting Proteins (DIP) [54] and methods described in the section False-Negative Rate from Literature. The true-positive rates from our capture–recapture model are in excellent agreement with rates estimated from overlap with the curated literature (Table 10). For yeast, the capture–recapture estimate is 16%, while the 95% confidence interval from the literature is 15%–20%; for fly, capture–recapture gives 10% and the literature comparison gives 6%–26%.

**Table 10:**
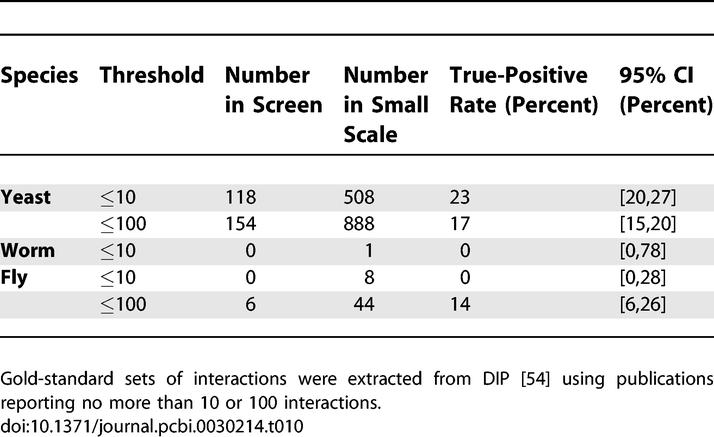
True-Positive Rates Estimated from Literature Comparisons

The capture–recapture method has two benefits over the literature comparison. First, for organisms with scant literature data, the literature comparison provides an uninformative broad range for the true-positive rate compared to the narrow range of the capture–recapture method. Thus, for worm, the capture–recapture method suggests a true-positive rate of 24% with a standard deviation of roughly ±3%, while the literature comparison gives a broad range of 0%–78% as a 95% confidence interval. Second, the capture–recapture method is able to identify independently the losses due to systematic factors and due to stochastic undersampling, while the literature comparison can only provide a lumped estimate.

### Comparison with Previous Total Interaction Count Estimates

The summary results, Table 3, extrapolate the number of interaction partners from the estimated number of true positives within the preys screened to the total number in the proteome. The results suggest about 40 pairwise interaction partners per protein in yeast, and roughly 100 pairwise interaction partners per protein in worm and fly.

These numbers, however, are based on the estimated means. For long-tailed degree distributions, the median values may provide greater intuition, and may in fact be more robust by discounting outliers with high interaction counts. Median numbers of interaction partners obtained from parametric degree distributions (see the section Total Interaction Counts), are provided at the bottom of Table 3. The final values obtained are roughly ten partners per yeast protein, 61 for worm, and 46 for fly. The 1.5-fold difference between worm and fly might point to built-in biases in the screens (different baits and preys, different selection thresholds, etc.) rather than any fundamental biological differences. Using the median and mean estimates as brackets, our results suggest between 30,000 and 140,000 pairwise interactions in yeast; 600,000 to 1,200,000 in worm; and 300,000 to 600,000 in fly.

Other work, using a contingency table approach similar to *k* ^∩^, has suggested a 95% confidence interval of about 40,000 to 75,000 interactions in yeast, and 150,000 to 370,000 in human [16]. This previous work was unable to make predictions for worm or fly, however, due to the lack of multiple datasets for comparison.

## Discussion

The methods introduced here provide a new model for false-positive and false-negative rates for two-hybrid screens. To our knowledge, this is the first model that considers the number of observations of each prey, as opposed to a binary interaction / no-interaction summary statistic, to calculate these rates. We have validated the model thoroughly using simulated data and using published biological datasets. The applications to published data demonstrate the crucial ability to predict how many new interactions will be observed as more preys are collected, together with the true-positive and false-positive fractions.

One of the major criticisms of the two-hybrid method has been a high false-positive rate. Unlike previous methods that produce average false-discovery rates over an entire screen, our method provides bait-by-bait estimates. False-discovery rates are heterogeneous: some baits perform better than others. As others have suggested [38], this permits the possibility of correlating false-positive rates with hydrophobicity and related protein properties. We find strong evidence for higher false-discovery rates for membrane proteins, but not for hydrophobic proteins in general. Two-hybrid screens such as the split-ubiquitin system [55] have been developed to detect interactions between membrane proteins. These assays could very well show a correlation of false-positive rates with other classes of proteins.

Classification of proteins according to enzymatic function reveals that those in signaling pathways have lower false-discovery rates than those in metabolic pathways. This suggests greater evolutionary pressure to maintain specificity of information-carrying networks.

One suggested mechanism of network evolution is that recent paralogs may continue to share interaction partners. This would imply that proteins within a single family should show cross-reactivity with each other's binding partners, eventually leading to false positives due to weak remnants of ancestral interactions. We rejected this hypothesis by finding no significant correlation between false-discovery rate and family size.

Analysis of false-positive rates also provides a quantitative estimate of the value of using constructs from a sequence-verified ORF collection rather than from cDNA libraries. When we classify bait constructs as “good” or “bad,” we find that the “good” category is 90% for an ORF collection and 45% for a cDNA library. On the prey side, using an ORF library produces one-third fewer false positives than a cDNA library.

This model yields estimates of false-negative rates from screening statistics, and to our knowledge is the first attempt to discriminate between false negatives due to undersampling and false negatives due to biological and systematic effects. We find that sampling and systematic factors are both important contributors to false negatives, with undersampling yielding a roughly 2× reduction in interactions, and systematic effects yielding an additional 2× to 6× reduction. False-negative rates estimated from the statistical model are in general agreement with those estimated from comparisons between datasets or to a gold standard.

The statistical framework provides a convenient route to assessing the likelihood of different population-level functional forms for the protein degree distribution and the false-discovery rate. We provide conclusive evidence that, among one-parameter degree distribution models, a PL model is far superior to an ER model. We find evidence for exponential truncation of the degree distribution in worm and fly, but not in yeast. The number of interactions per protein is predicted to increase from about ten partners for yeast to about 50 partners for the metazoans worm and fly. These results suggest that more complex organisms have more interactions per protein component, as well as more components overall.

This model will have value in application to ongoing pool-based assays for protein–protein interactions in model organisms and human. An immediate demonstration is the ability to predict the total number of pairwise protein–protein interactions based on two-hybrid data. We suggest that the total number of pairwise interactions observable by the two-hybrid system is roughly 140,000 in yeast, and 600,000 to 1,300,000 in worm and fly, with about 95% remaining to be discovered.

An attractive extension of the model presented here is to include unequal capture probabilities for true interaction partners. The current model represents true-positive preys as a two-component mixture: a fraction 1 − *p*_syst_ of true-positive preys are considered absent from the pool, with capture probability 0; the remaining *k* true-positive preys have uniform capture probability. It would be possible to include more components, or even a continuous variable representing an inhomogeneous capture probability of a prey. This is important for libraries generated directly from mRNAs with varying abundances, and could still be important for libraries generated from normalized clone collections due to varying effective nuclear concentrations and binding constants.

Including heterogeneous capture rates for baits could be accomplished by extending the model to represent the true-positive rate *p*_syst_ for each bait as an additional hidden variable to be optimized within the Expectation–Maximization (EM) framework. In this work, *p*_syst_ is a global parameter calculated after the sampling-based parameters have been estimated.

Both of the above extensions would involve a probability model that considers interactions in both directions, bait–prey and prey–bait, and would necessarily add complication to what is already a mathematically detailed model. While a more complicated model would seem unlikely to lead to different conclusions from those presented here, it could answer questions relating capture probabilities to protein physical properties, protein abundances in libraries, and transient versus stable protein interactions.

Developing related statistical models for other types of protein interaction screens will also be important. A constant proviso attached to interaction screens is the suspicion that methods such as two-hybrid screens, affinity pull-downs [56,57], and protein binding chips [58] will identify different subsets of interactions. Quantitative comparisons are difficult, however, because systematic assay-specific differences are confounded with random loss of interactions due to incomplete sampling. Methods such as the one presented here will contribute to understanding what different screening technologies tell us about the proteome.

## Materials and Methods

### Theory.

An overview of notation is provided (Table 1). Consider a particular protein *j* used as one of *N* baits in a two-hybrid screen against a pool of Γ species of preys of which *κ_j_* are true interaction partners. We assume that *κ_j_* = Γ so that Γ − *κ_j_* ≈ Γ is a good approximation for the number of true negatives for each bait. We model the first stage of a two-hybrid screen as an all-or-none process reflecting whether a bait mates successfully with a prey and yields progeny that survive selection. For simplicity, and to reduce the number of free parameters, we assume an identical systematic true-positive rate *p*_syst_ for each bait *j* with each of its *κ_j_* true interaction partners. The parameter *p*_syst_ includes systematic biological effects, such as generating functional fusion proteins in the two-hybrid system. The number of surviving true positives is *k_j_* with binomial distribution

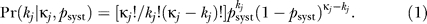



We further assume that the Γ true negatives continue to grow slowly in the selective media with a population expansion that is only *p* ^*^ times the population expansion of surviving true positives. We assume a stochastic, not systematic, model for false positives, with *p* ^*^ = 1 and identical for each prey.

Also for simplicity, and in accord with prey libraries constructed from normalized ORF collections, we assume that each prey is present initially at equal concentrations. The final mass fraction of false positives is denoted *α_j_* = *p* ^*^Γ[*k_j_* + *p* ^*^Γ], yielding a scaled error model that depends on a single constant *p* ^*^Γ ≡ *a*. More generally, a variety of error models are possible:

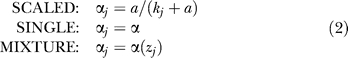



The MIXTURE model introduces an index *z_j_* ∈ {1,2…,*m*} to one of *m* possible values of *α* and prior probabilities *π*(1) + *π* (2) + …+ *π* (*m*) = 1 for the *m* components. With *m* = 2, this permits “good” baits (*z_j_* = 1) and “bad” baits (*z_j_* = 2) with *α*(1) ≤ *α*(2).

The second stage of screening bait j is to sequence *n_j_* clones from the mixture of true positives and false positives. We assume that each of the *k_j_* true positives is sampled with uniform probability (1 − *α_j_*)/*k_j_* and each of the Γ false positives is sampled stochastically with uniform probability *α_j_*/Γ. The number of times that prey species *m*, either a true positive or a false positive, is sampled within the *n_j_* clones is *n_jm_*, with 0 ≤ *n_jm_* ≤ *n_j_* and ∑*_m_n_jm =_ n_j_*. The probability of the observed counts *n_jm_* is a multinomial, [*n_j_*!/Π*_m_n_jm_*!]Π*_m_* 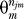 , with *θ_m_* = (1 − α*_j_*)/*k_j_* or α*_j_*/Γ.

As is typical in a capture–recapture setting, it is more convenient to work in the context of abundance classes. Let 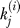 for *i* ≥ 1 represent the observed data as the number of preys observed exactly *i* times within the *n_j_* samples. For convenience, we introduce *s_j_* as a synonym for 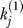 , the number of singleton preys observed only once. The total number of distinct preys observed is *w_j_*,

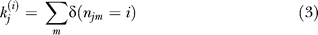


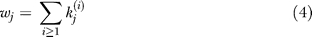


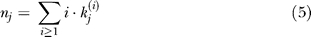
where δ(arg) is 1 if its argument is true and 0 if false. The standard generalized multinomial distribution is obtained by summing over the {*S*} permutations that yield *w_j_* distinct species,

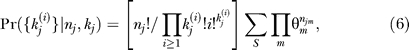
identical to Equation 3 of [34]. A rough motivation for this formula is that *n_j_*/Π*_i_*_≥1_ 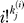 is the number of distinct permutations of the *n_j_* clones, |*S*|/Π*_i_*_≥1_ 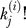 is the number of distinct permutations of the observed species, and Π*_m_* 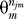 is the probability of selecting the species in specified order.

Our final approximation is that each true negative occurs at most once as a false positive, *n_jm_* = 0 or 1 when *m* is a true negative. The expected number of false-positive clones within the *n_j_* clones is *αn_j_*. The probability that each of these, selected at random from the Γ total possibilities, is distinct is 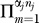 [1 − (*m* − 1)/Γ], or approximately exp[−(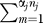 *m* − 1)/Γ] = exp[−α*_j_n_j_*(α*_j_n_j_* − 1)2Γ] , analogous to the Birthday Paradox (the probability that two people in a large random group share a birthday). An appropriate constraint ensuring distinct false positives is that *n_j_* ≤ 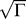 /α*_j_*. With genome-size prey libraries, Γ ≥ 5,000, and we anticipate that *α_j_* ≤ 0.5, making this approximation valid for *n_j_* ≤ 140. For yeast, ten baits (0.67%) violate this constraint; for worm, 18 baits (2.5%); for fly, ten baits (0.27%).

Denote *f_j_* as the number of false-positive observations within the sample *n_j_*. By the above assumption, the false positives must be within the *s_j_* singletons, and 0 ≤ *f_j_* ≤ *s_j_*. Using the uniform capture probabilities, Π*_m_* 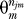 = 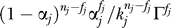 . The number of permutations |*S*| can be calculated under the above assumption of singleton false positives as

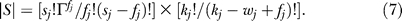



The first factor is the number of ways that false positives can be assigned to a subset of *f_j_* of the *s_j_* singleton species. We have used Γ!/(Γ − *f_j_*)! ≈ 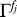 , which is valid because Γ ≫ *n_j_* ≥ *f_j_*. The second factor is the number of permutations that select the *w_j_* − *f_j_* observed true positives out of *k_j_*. Combining results yields

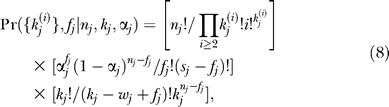
with an additional factor of *π*(*z_j_*) depending on the hidden variable *z_j_* that indicates the component for the MIXTURE error model, Equation 2.

The probability distribution for the hidden variables *f_j_* and *k_j_* are obtained through the Bayesian relation

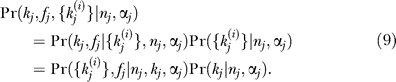



For the MIXTURE model, the analogous equation includes the hidden variable *z_j_*. When *k_j_* is independent of *n_j_* and *α_j_*, Pr(*k_j_* | *n_j_*,*α_j_*) = Pr(*k_j_*) ≡ Pr(*k_j_* | Φ), where Φ comprises one or more global parameters describing the interaction degree distribution. The simplified Bayesian result is

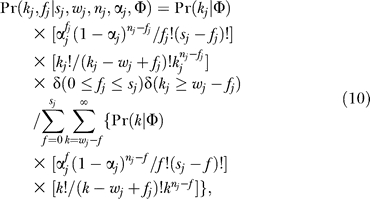
or Pr(**y** *_j_* | **x** *_j_*,**Q**) where the hidden variables **y** *_j_* = { *k_j_*, *f_j_*}, and possibly *z_j_*; the observed variables **x** *_j_* are the counts of singletons (*s_j_*), distinct preys (*w_j_*), and total samples (*n_j_*); and the parameters **Q** are the global parameters for the error model (*a*, *α*, or {α(1)… *α*(*m*);*π*(1)…*π*(*m*)}) and the protein degree distribution. The three summary statistics {*s_j_*, *w_j_*, *n_j_*} are sufficient statistics for the observed data 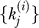 due to the assumption of homogeneous probabilities for observing each true-positive and true-negative species. The sum over *k* formally starts at *w_j_* − *f*, which may equal 0 when each of the *n_j_* observations is a singleton. In the results, however, we restrict attention to probability distributions for which Pr(*k* = 0 | Φ) = 0 and start the summation at *k* = 1.

Three distributions are considered:

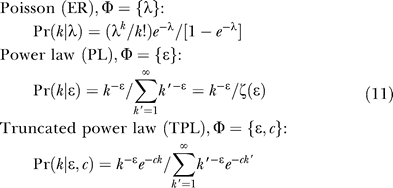



The normalization of the Poisson distribution by 1 − *e* ^−λ^ in Equation 11 reflects that the summation begins at *k* = 1 rather than at 0.

In keeping with the definition of 1 − *p*_syst_ as the systematic false-negative rate, it may be more appropriate to use parametric distributions for Pr(*κ*), than to obtain Pr(*k*) as the convolution Pr(*k*) = ∑*_κ_*_≥k_ Pr(*k* | *κ*)Pr(*κ*). We are in a sense replacing Pr(*k* | *κ*) of Equation 1 by a delta function near the mean value *κp*_syst_ in order to retain the form of a simpler parametric distribution.

### Parameter estimation.

Estimates for {*k_j_*, *f*} for each bait could in principle be obtained using Equations 10–11. This requires, however, estimates for the global parameters **Q**. Furthermore, the asymptotic form of the summand in Equation 10 is 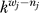 Pr(k | Φ). Writing the asymptotic form of Pr(*k* | Φ) as *k* ^−*ɛ*^, existence of a maximum a posteriori estimator requires *n_j_* + *ɛ − w_j_* > 0; convergence of the sum requires *n_j_* + *ɛ − w_j_* > 1; and convergence of the mean of *k_j_* requires *n_j_* + *ɛ − w_j_* > 2. For the ER prior, *λ* > 0 guarantees convergence of all powers of *k_j_*; for the TPL prior, *c* > 0 guarantees convergence. Convergence could be achieved by normalization of *k* in Equation 11 to a finite cutoff Γ rather than to ∞. In practice, however, results for the PL model are sensitive to the cutoff value when *ɛ* < 2. The TPL and ER models are not sensitive to a cutoff, as both provide a natural cutoff as part of the model parameters. If a cutoff is appropriate, we anticipate that these models will provide improved descriptions of a degree distribution.

To overcome both these difficulties, we use EM to obtain parameter estimates **Q̂** that maximize the probability of the observed data [59,60],

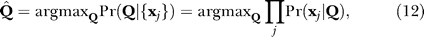
assuming a uniform prior Pr(**Q**). We introduce the notation

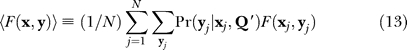
for the mean of a generic function *F*(**x**,**y**) of the hidden and observed variables. The sum over the hidden variables expands to

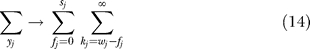
for the scaled *α* and single *α* error models, and to

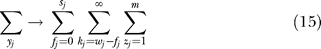
for the *m*-component mixture. As mentioned before, while some models permit *k_j_* = 0, the power law model does not; for consistency, we start all degree distributions at *k_j_* = 1 and the lower limit of the sum over *k_j_* is effectively max(1,*w_j_* − *f_j_*). The standard equations giving a new parameter estimate **Q** in terms of a previous estimate **Q**′ are

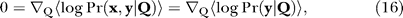



where the simplification holds because Pr(**x** *_j_*,**y** *_j_* | **Q**) = Pr(**x** *_j_* | **y** *_j_* , **Q**) Pr(**y** *_j_* | **Q**) and Pr(**x** *_j_* | **y** *_j_*, **Q**) = Pr(**x** *_j_* | **y** *_j_*) is independent of **Q**. Update equations for the error models are as follows:

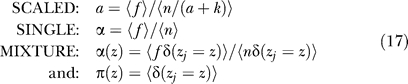



Update equations for the degree distribution are as follows:

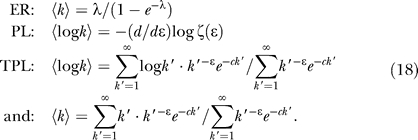



An interesting and unfortunately common boundary case occurs when only a single clone is sequenced for a bait, *n_j_* = 1. In these cases, *s_j_* and *w_j_* must also be 1, and Pr(**x** *_j_* | **Q**) = 1 regardless of **Q**. Thus, baits with *n* = 1 do not affect the final model parameters because the partial derivatives of their contributions to the log-likelihood are always 0.

The appearance of the expectation of log *k* rather than *k* in the EM equations for the power law parameter *ɛ* in the PL and TPL models suggests the use of the posterior mean of log *k* as a route to estimating the hidden variable decay. We define this estimator as **k̂**,

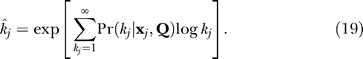



### Model selection.

The three error models and the three degree distribution models yield a total space of nine possible models, with varying degrees of freedom (df): 1 df for the scaled and single error models; 2*m* − 1 df for the *m*-component mixture error model; 1 df for the ER and PL degree distributions; 2 df for the TPL distribution. We used three separate criteria to assess which model provides the best fit: log-likelihood cross-validation (CV); full data Bayesian information criterion (BIC); and bootstrap BIC.

The CV method with *F*-fold cross-validation divides the full data into *F* subsets. For subset *f*, model parameters **Q** *_f_* are estimated using the remaining *F* − 1 subsets, and the log-likelihood of subset *f* is calculated as loglik*_f_* = log Pr({**x** *_f_*}|**Q** *_f_*). This procedure is repeated *F* times, once for each subset. Thus, each subset is used *F* − 1 times to obtain model parameters and 1 time to obtain an unbiased log-likelihood. The final log-likelihoods, ∑*_f_* loglik*_f_*, can be compared directly. The statistical significance of a difference in log-likelihoods for two models can be assessed using a paired test, such as the nonparametric Wilcoxon rank signed test, for the differences 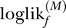 − 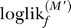 for pairs of models *M* and *M′*.

The BIC is an appropriate heuristic for performing model selection in the context of maximum likelihood parameter estimation for **Q** and a uniform prior over model classes *M*:

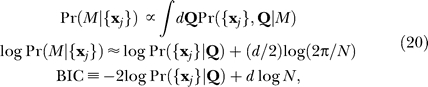
where *d* is the number of df in the model and *N* is the number of sets of observations, here baits. A smaller BIC indicates a more likely class of models, and the term *d* log *N* penalizes more complex models. Overfitting is unlikely for our models: the typical number of sets of observations *N* ∼ 1,000, while the models have only two to four free parameters.

Sometimes, the BIC heuristic may indicate a small preference for one model over another. Bootstrap replicates may be used to assess the stability of the BIC results. Bootstrap replicates are constructed by selecting *N* examples from the full data of *N* examples uniformly and with replacement. Thus the number of times *n* that an example occurs in a bootstrap replicate is approximately Poisson with Pr(*n*) = 1/(*n*!*e*). The BIC heuristic for each model is then calculated for each bootstrap replicate, and the number of times that each model has the best BIC score is recorded.

### Domain-specific false-positive rates.

We calculated the cumulative number of clones sampled for a domain, *n*_dom_, and the cumulative posterior estimate for the number of false positives, **f̂**_dom_, by summing over the counts for each protein annotated as having that domain:

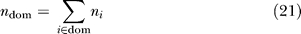


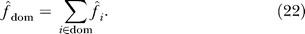



*p*-Values for the upper and lower tail, *p*_>_ and *p*_<_, were calculated assuming a binomial distribution with *n*_dom_ trials and a success rate equal to the overall false-discovery rate α̂ for each organism (0.093 for yeast, 0.122 for worm, 0.157 for fly). To ensure a conservative test, fractional values of **f̂**_dom_ were rounded down for the upper-tail test and rounded up for the lower-tail test,

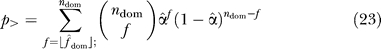


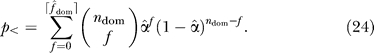



Finally, the single-value *p*-values were adjusted for the number of domains observed among baits in each species (783 for yeast, 473 for worm, 1,310 for fly). When two domains refer to an identical subset of proteins, results for only a single domain are displayed.

### Validation with simulated data.

*Parameter estimates.* Simulations were performed separately for each of the three protein degree distributions (ER, PL, TPL) combined with each of the three error models (scaled, single, mixture), yielding nine total models. Simulations over a range of parameter values used *N* = 1,000 baits and *n* = 10 clones sampled per bait, and were usually repeated three times at each parameter value (Figures S1–S3). The agreement between known and estimated parameters is very good over parameter values that span the estimated values for the published datasets (Table S1). Agreement is quantified by the root-mean-square (RMS) difference between the known and estimated parameters and the *R* ^2^ goodness of fit measure, defined as 1 − ∑*_t_*(θ*_t_* − θ̂*_t_*)^2^/∑*_t_*(θ*_t_* − θ̄)^2^ with sum *t* over trials, θ*_t_* the true parameter value for trial *t*, θ̄ the mean of θ over the trials, and θ̄*_t_* the estimated value for trial *t*. The RMS values are generally less than 0.05 in absolute units. The *R* ^2^ values for the TPL-mixture range from 0.65 for the false-discovery rate parameter to 0.98 for the power law parameter. The *R* ^2^ values depicted for the TPL-mixture model are not typical but rather the worst results obtained over all combinations of degree distribution and error model (Figure S1–S3). Other models with fewer parameters are more accurately estimated. For example, simulations using the PL-mixture model yield an *R* ^2^ of 0.99 for *ɛ*, 0.97 for *π*(1), and 0.88 for *α*(1) and *α*(2). Simulations using the PL-single model and PL-scaled model yield *R* ^2^ ≥ 0.99 for all parameters (Table S1).

*Hidden variable estimates.* Simulations also assessed the ability to predict hidden variables (Figure S4). We simulated a dataset using parameters obtained by fitting the fly experimental data with a TPL degree distribution and a two-component error model. The simulated dataset had *N* = 1,000 baits and *n* = 10 baits per prey. Next, the EM algorithm was used to estimate the model parameters ɛ̂, α̂(1), α̂(2), and π̂(1). The estimated parameter values, rather than the true known values that generated the data, were used to avoid introducing a favorable bias in the hidden variable predictions. The converged model parameters were then used to obtain the posterior estimates 〈log *k*〉, 〈*f*〉, and 〈*δ* (z = 1)〉 for each bait, with the notation 〈…〉 defined by Equation 13 (Figure S4). Using the log-transform of *k* is more natural than using *k* due to the long tail of the power law distribution. It is also motivated by the EM equations for the power law exponent *ɛ*, which depends on 〈log *k*〉 rather than 〈*k*〉 as shown in Equation 18.

The hidden variable 〈log *k*〉 is estimated with *R* ^2^ = 0.84, indicating good correlation between true and estimated values. The RMS error in the estimate is 0.42, indicating the ability to predict the true value of *k* within a factor of exp(±0.42), or 1.5-fold. The RMS for the number of false positives is 1.1, which means that the estimate for the number of false positives for a bait is usually within 1 of the true count. Estimates of the error component of a bait are accurate for low values (low error rate) and high values (high error rate) of **ẑ**. Baits with intermediate estimated values, 1.2 ≤ **ẑ** ≤ 1.6, may come from either error component. For these baits, all but one or two of the preys are singletons, and it is difficult to determine whether this is due to a large bait degree or a large error rate.

*Model selection for simulated data*. We next validated the BIC heuristic for model selection (Table S2). In this test, we used each of the nine possible models (three degree distributions × three error models) to generate datasets with *N* = 1,000 baits and *n* = 10 preys per bait, then calculated the log-likelihood for each of the nine models. A total of 81 fits were performed (nine generative models × nine fitting models). The parameters of the generative models were deliberately selected to yield similar data by using the values obtained by fitting the experimental worm data (Table 2). In each case, the BIC identified the model accurately (Table S2). The probability to obtain a perfect result by chance is approximately (1/9)^9^, or 2.6 × 10^−9^.

The BIC results indicate the TPL model can provide a good fit for data generated by all three models: ER, PL, and TPL. The TPL model includes the PL model as a special case with the exponential decay constant *c* = 0. A large value of *c*, which truncates the degree distribution, permits the TPL model to mimic the ER model. The BIC score adds a penalty of log 1,000 = 6.91 to the TPL fit to account for the extra parameter. In several of the entries of Table S2, this penalty is essential to select the true generative model over the TPL model.

### Model selection for experimental data.

Properties of the experimental datasets for yeast, worm, and fly are summarized at the top of Table 2: total number of baits, *N*; number of preys sampled per bait, *n*; number of unique observed interaction partners per bait, *w*; and number of interaction partners observed a single time, *s*. Each dataset was fit using three separate degree distribution models (ER, PL, TPL) and three error models (SCALED, SINGLE, MIXTURE), a total of nine possible combinations of degree distribution and error model.

*Model selection.* The BIC heuristic selects the PL-MIXTURE model for yeast and the TPL-MIXTURE model for worm and fly (Table 2). In general, the PL and TPL models are much better than the ER models. The MIXTURE error model is somewhat better than the SINGLE error model, which in turn is much superior to the SCALED error model. To explore the robustness of this conclusion, we used 10-fold cross-validation (CV) to compute the log-likelihood of the data under each of the models. The CV method identifies the TPL-MIXTURE model as the best for worm and fly, and finds no significant difference between PL-MIXTURE and TPL-MIXTURE for yeast (*p*-value = 0.35). Finally, we generated 100 bootstrap replicates each of the yeast, worm, and fly datasets, calculated the BIC scores for each of the nine models, and tabulated the number of times that each model had the best score. The PL-MIXTURE model won 94/100 times for yeast, and the TPL-MIXTURE won 98/100 times for worm and 100/100 times for fly.

*Model parameters.* The PL models yield power law parameters *ɛ* that are robust to the choice of error model: *ɛ* = 1.67–1.72 for yeast, 1.48–1.53 for worm, and 1.50–1.58 for fly. In contrast, we can mimic a conventional fit by estimating the PL *ɛ* using Equation 18 but with *k* (the corrected number of interaction partners) replaced with *w* (the observed number of unique partners, which may include false positives). The conventional fit introduces two sources of error: it inflates bait degrees by including false positives, and it deflates bait degrees by excluding false negatives. The conventional estimates for yeast, worm, and fly yield exponents of 2.22, 1.66, and 1.61, which are larger (have steeper decay of the degree distribution) than the model results. Thus, the error due to false negatives may dominate the error due to false positives when PL parameters are estimated. Parameter estimates based on prey degree rather than bait degree might be less sensitive to these sources of error.

Work by others connects the inverse of *c* to the typical domain size in a network [15]. The best-fitting TPL models for worm and fly have *c* ≈ 0.04 to 0.07, suggesting a domain size of ten to 30 proteins in a subnetwork. These estimates may not be overly precise as the TPL parameters are sensitive to the error model. The worm data, for example, yields (*ɛ*, *c*) = (1.25, 0.012) for scaled, (0.46. 0.035) for single, and (0.95, 0.040) for mixture error models. The extra variability arises because a larger value of *ɛ* can compensate for a smaller value of *c*. The yeast network, which is best fit by a PL-MIXTURE model, yields a very small value of *c* when fit by any TPL model. This suggests that the yeast network may show less modular structure than either the worm or fly networks. Interaction networks from viruses, parasites, and simpler organisms have been shown to be less clustered than interaction networks from more complex organisms [52].

The fraction of false positives is estimated consistently by the SCALED, SINGLE, and MIXTURE error models regardless of the choice of degree distribution. The false-positive fraction is calculated as ∑*_i_*f̂*_i_*/∑*_i_n_i_* from the estimated false-positive count **f̂*_i_* and number of preys *n_i_* for each bait *i*. This fraction is estimated as 0.08–0.09 for yeast, 0.12 for worm, and 0.16 for fly. The false-positive fraction is, of course, larger when defined relative to the number of unique interactions identified rather than the number of preys.

### Cross-validation with experimental data.

Although the generative model, the model parameters, and the hidden variables are unknown for the experimental yeast, worm, and fly data, cross-validation is still possible. The cross-validation used half of the dataset to predict the number of new and single interactions in the remainder of the dataset.

First, for each bait, we extracted a random half of the preys to serve as a training set. For baits with an odd number *n* of preys, we selected (*n* − 1) / 2 and (*n* + 1) / 2 for the training set with equal probability. Next, we used the training half of the data to estimate model parameters for each organism. For simplicity, we restricted attention to TPL–MIXTURE model.

With the maximum likelihood parameter estimates, we then obtained posterior estimates for the false-positive rate and true interaction count of each bait protein. The false-positive rate was determined directly from the mixture model. Based on experience with simulated data, we used exp(〈log *k*〉) for the posterior estimate of the bait degree. As noted before, the EM equations for PL-like networks, Equation 18, suggest that the logarithm of the degree is a more natural variable than the degree itself (which is not even guaranteed to converge).

Finally, we use the statistical model to predict how many unique interaction partners and singleton interaction partners are expected to be observed as the remaining test half of the data is added back. The predictions of the model based on the training half can then be compared with the actual results for the number of unique partners, *w*, and the number of singleton partners, *s*. Since the posterior estimate of the bait degree may be fractional, we replaced the factorial function with the Gamma function. Also, rather than starting with the values for *s* and *w* for the training set and calculating the marginal increase, we performed a more demanding comparison by using the model parameters to estimate *s* and *w* for the observed training set as well. Fitting error was observed only for the count of yeast singletons.

### False-negative rates.

Our model can distinguish between random or stochastic false negatives due to undersampling, which could be detected by sampling additional clones, and systematic false negatives that cannot be corrected by deeper sampling of clones from a two-hybrid screen. As described in the section Theory, the overall true-positive rate is the product of the random and systematic rates, *p*_samp_ × *p*_syst_.

The true-positive rate from sampling for bait *i* is (*w_i_* − *f_i_*) / *k_i_*, the number of true positives observed out of the *k_i_* true positives represented in the pool. The values of *f_i_* and *k_i_* are hidden, however, and *p*_samp_ must be estimated. An appropriate estimator, weighting each true positive equally, is

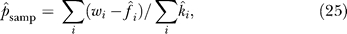
using Equation 19 to define **k̂*_i_*.

The false-negative rate due to systematic losses may be estimated from interactions observed in both directions. We first restrict attention to proteins that were found to have at least one interaction as a bait and as a prey. Using the notation that *n_ij_* indicates the number of times that bait *i* recovers prey *j*, we extract the subset with *n_ij_* ≥ 2, ensuring that the interaction between *i* and *j* is a true positive. These *N*_2_ cases are then subdivided into *N*_0_ not observed in the reverse orientation, with *j* as bait and *i* as prey, and *N*_+_ observed at least once in the reverse orientation:

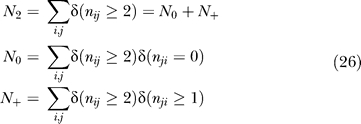



The indicator function *δ*(arg) is 1 for a true argument and 0 for a false argument. The expected value of *δ*(*n_ji_* ≥ 1) is the true-positive rate for bait *j*, equal to the product of the true-positive rates accounting for systematic losses and for random undersampling,

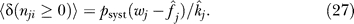



Similarly, the expectation of *δ*(*n_ji_* ≥ 0) is 1 − *p*_syst_(*w_j_* − **f̂*_j_*)/**k̂*_j_*. The estimated true-positive rate from just the sampling step for the interactions contributing to *N*_2_, denoted 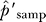 , is

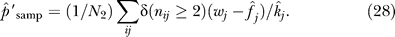



In practice, we find that 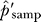 is somewhat larger than the value of (*w* − **f̂**)/**k̂** averaged over all baits. For yeast, worm, and fly, the values for 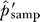 are (0.81, 0.84, 0.81), and the values for 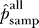 averaged over all baits are (0.58, 0.60, 0.71). Note that 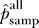 , the average of the ratios, is distinct from **p̂**_samp_, the ratio of the averages of *w* − **f̂** and **k̂** (Table 10).

An estimator for *N*_+_ in terms of the unknown *p*_syst_ and other quantities that are known is

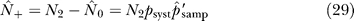



An obvious route to estimating *p*_syst_ is to assume that *N*_+_ follows a binomial distribution for *N*_2_ trials with success rate 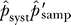 . The corresponding maximum likelihood estimate **p̂**_syst_ and its standard error are

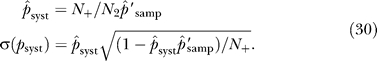



The estimated number of true interaction partners corrected for sampling losses and systematic losses is then κ̂*_i_*, with

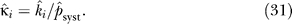



### False-negative rate from literature.

An alternative estimate for the false-negative rate of a high-throughput screen may be obtained by comparison to literature data. To accomplish this, we downloaded protein interaction datasets from the Database of Interacting Proteins (DIP) [54]. As is common in studies such as this, we required a “gold-standard” dataset that did not use information from the two-hybrid screens we are studying and also was unlikely to be contaminated by false positives. We therefore filtered the entire DIP database to include interactions from only small-scale experiments. Because there is no firm definition of small scale, we used cutoffs of 10 and 100 for the number of interactions reported.

For each unique gold-standard interaction, denote the pair of proteins (*i,j*). We defined *b_i_* = 1/0 if protein *i* was / was not used as a bait in the high-throughput screen, and *p_i_* = 1/0 if protein *i* was / was not used as a prey. We defined similar terms for *b_j_* and *p_j_*. Also define *I*(*i*, *j*) as 1/0 if the high-throughput screen detected or missed the interaction between *i* as bait and *j* as prey. We then calculated the following values:

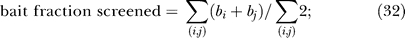


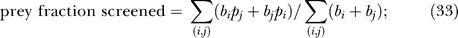


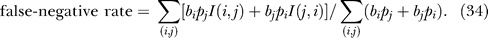



These summary statistics consider the gold-standard interaction in both orientations. For gold-standard interactions between identical proteins, only one of the two identical terms was included in the sum. The bait fraction screened and the prey fraction screened indicate possible correlation in the choice of baits and preys in the small-scale experiments and the high-throughput screens. The summary statistic for the false-negative rate should correct for this bias by only considering interactions that were within the space considered by the high-throughput experiment. This false-negative rate is interpreted as including both the undersampling loss and the systematic loss.

### Total interaction counts.

The mean number of true interaction partners per bait, corrected for false positives and for undersampling, is

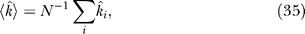



Using the definition of the false-negative rate from undersampling, *p*_samp_ from Equation 25, the mean may also be written as

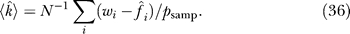



Correcting the mean for systematic losses, *p*_syst_ from Equation 30 , yields

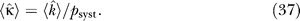



This value requires a final correction for the actual search space relative to the entire genome size. The correction we use is the number of preys with at least one interaction, *N*_prey_, relative to the number of proteins annotated in the entire genome, *N*_proteome_. The final proteome-wide mean interaction count is (*N*_proteome_/*N*_prey_)〈κ̂〉. The number of pairwise interactions in the entire proteome is then

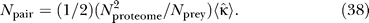



An alternative method for estimating interaction counts is to use the inferred probability distribution directly: Pr(*k* | *λ*) for Poisson; Pr(*k* | *ɛ*) for PL; and Pr(*k* | *ɛ*, *c*) for TPL, Equation 11. For brevity, denote each of these Pr(k | θ̂) for the appropriate parameter estimate θ̂. An overall value for *k* denoted k̂ may be obtained using an analog of the bait-specific estimate Equation 19,

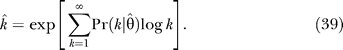



A more typical value for the interaction count corrected for undersampling may be obtained using the median defined by the parametric distribution. We use linear interpolation to estimate a non-integer median,

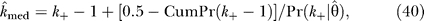
where *k*_+_ is the smallest value of *k* such that CumPr(*k*) ≥ 0.5 and CumPr(*k*) is the cumulative probability 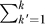 Pr(*k*′ | θ̂) The median interaction count corrected for systematic losses, κ̂_med_, is then

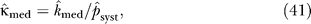
and the median number of interaction partners per protein, corrected for the space screened, is (*N*_proteome_/*N*_prey_)κ̂_med_.

## Supporting Information

Dataset S1 Raw Data, Parameter Estimates, and Hidden Variable Estimates for Yeast, Worm, and Fly Two-Hybrid Screens (273 KB TXT)

Dataset S2 Raw Data and Comparison of Interaction Count Estimators for Yeast Proteins (72 KB TXT)

Figure S1 Parameter Estimates for Simulated Datasets Using *N* = 1,000 Bait Proteins; *n* = 10 Preys Sampled per Bait; Erdös-Rényi Protein Degree Distributions with Mean Degree *λ*; and SCALED (Top), SINGLE (Middle), and MIXTURE (Bottom) Error Models The MIXTURE error model has two components with population fractions *π*(1) and *π*(2) = 1 − *π*(1) and false-discovery rates *α*(1) and *α*(2). Estimated parameter values are depicted for three independent trials.
(42 KB PDF)

Figure S2 Same as Figure S1 but for Power Law Degree Distributions with Power Law Exponent *ɛ*
(71 KB PDF)

Figure S3 Same as Figure S1 but for Truncated Power Law Degree Distributions with Power Law Exponent *ɛ* and Exponential Decay *c*
(55 KB PDF)

Figure S4 Estimated Values of Hidden Variables Compared to Known Values for the Bait Degree *k* (Left), Number of False-Positive Preys *f* (Middle), and Mixture Model Error Component *z* (Right) for a Dataset Simulated Using Parameters Obtained from a Truncated Power Law, Mixture Model Fit of the Fly Experimental Data (117 KB PDF)

Figure S5 Cumulative Degree Distributions Displayed for the Raw Number *w* of Unique Interaction Partners; the Estimated Number κ̂ from Equation 19; and from a Previous Estimator κ̂^∩^[22] The steps observed in κ̂ are from baits with *n* = *w* = *s*, with values provided in Table S6.
(70 KB PDF)

Table S1 Parameter Value Ranges and RMS and *R* ^2^ for Parameter Estimates Are Provided for Simulated Datasets (164 KB DOC)

Table S2 BIC Scores Obtained by Simulating Datasets by Each of Nine Generative Models and Calculating the Likelihood of Each Dataset under the Same Nine Models Degree distributions are Erdös-Rényi (ER), power law (PL), and truncated power law (TPL). Error models are scaled *α* (SCALED), single *α* (SINGLE), and a two-component mixture (MIXTURE). Parameter values for the generative models are based on the fit values for worm (Table 2). The simulations used *N* = 1,000 baits and *n* = 10 preys per bait.
(52 KB DOC)

Table S3 Cellular Component Gene Ontology Terms Whose Baits Have a False-Discovery Rate Significantly Different from the Organism Mean (5 KB TXT)

Table S4 Biological Process Gene Ontology Terms Whose Baits Have a False-Discovery Rate Significantly Different from the Organism Mean (20 KB TXT)

Table S5 Molecular Function Gene Ontology Terms Whose Baits Have a False-Discovery Rate Significantly Different from the Organism Mean (7 KB TXT)

Table S6 Estimates **k̂** and **f̂** Provided for Baits in Which Every Recovered Prey Was Unique (97 KB DOC)

## Abbreviations

BIC: Bayesian information criterion
CV: cross-validation
df: degrees of freedom
EM: Expectation–Maximization
ER: Erdös-Rényi
PL: power law
TPL: truncated power law
