# Supplementary material for: Where Have All the Interactions Gone? Estimating the Coverage of Two-Hybrid Protein Interaction Maps
Source: PLoS Comput Biol. 2007 Nov 23;3(11):e214. doi: 10.1371/journal.pcbi.0030214 (PMC2082503; doi:10.1371/journal.pcbi.0030214)
Supplement: Supplementary file 1 [file pcbi.0030214.sg001.pdf]

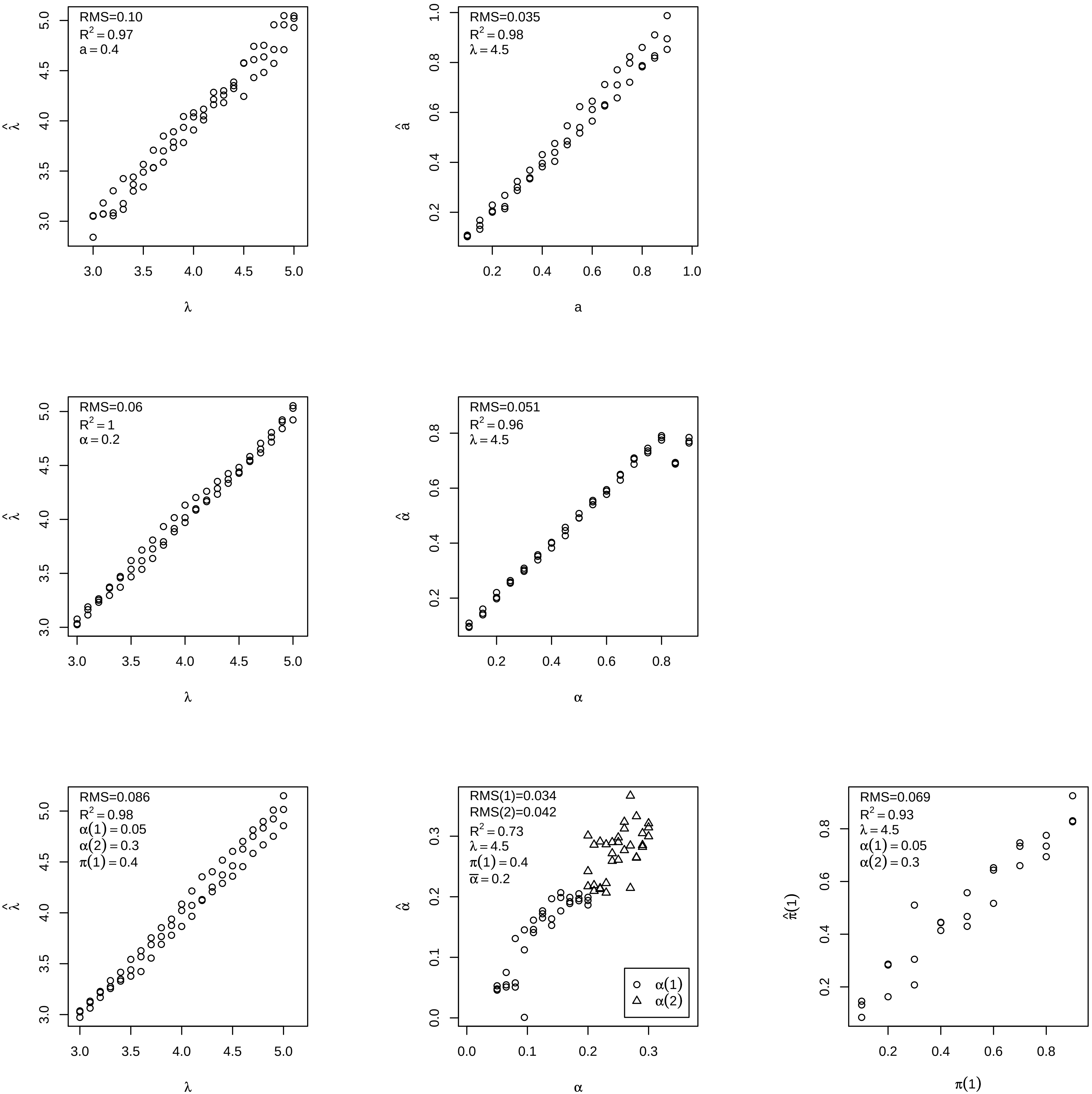

RMS=0.10
R2 = 0.97
a = 0.4
λ
λ̂
RMS=0.035
R2 = 0.98
λ = 4.5
a
â
RMS=0.06
R2 = 1
α = 0.2
λ
λ̂
RMS=0.051
R2 = 0.96
λ = 4.5
α
α̂
RMS=0.086
R2 = 0.98
α(1) = 0.05
α(2) = 0.3
π(1) = 0.4
λ
λ̂
RMS(1)=0.034
RMS(2)=0.042
R2 = 0.73
λ = 4.5
π(1) = 0.4
ᾱ = 0.2
α(1)
α(2)
α
α̂
RMS=0.069
R2 = 0.93
λ = 4.5
α(1) = 0.05
α(2) = 0.3
π(1)
π̂(1)
