# Supplementary material for: Where Have All the Interactions Gone? Estimating the Coverage of Two-Hybrid Protein Interaction Maps
Source: PLoS Comput Biol. 2007 Nov 23;3(11):e214. doi: 10.1371/journal.pcbi.0030214 (PMC2082503; doi:10.1371/journal.pcbi.0030214)
Supplement: Supplementary file 2 [file pcbi.0030214.sg002.pdf]

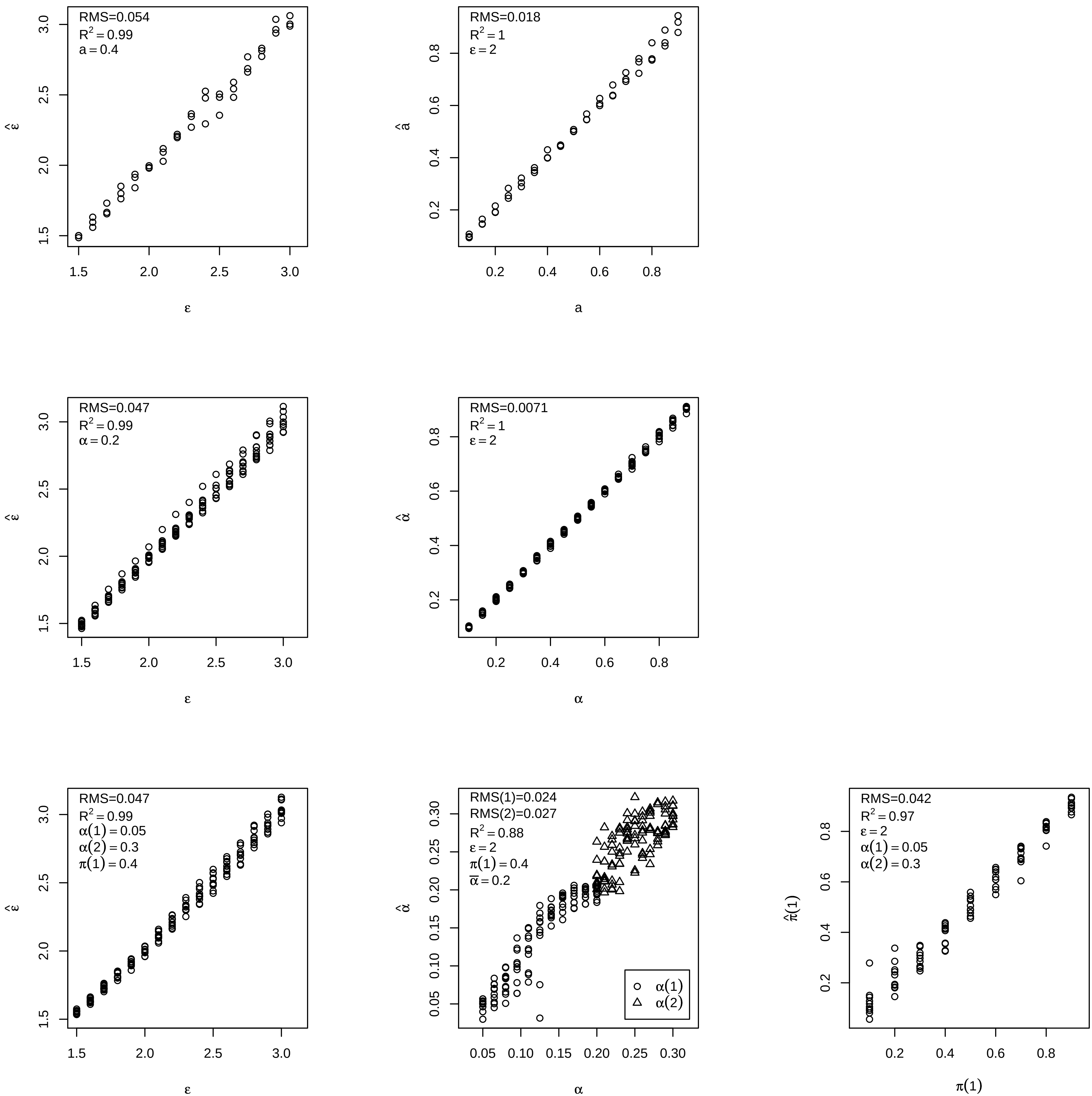

RMS=0.054
$R^2 = 0.99$
a = 0.4
$\hat{\varepsilon}$
ε
RMS=0.018
$R^2 = 1$
ε = 2
$\hat{a}$
a
RMS=0.047
$R^2 = 0.99$
α = 0.2
$\hat{\varepsilon}$
ε
RMS=0.0071
$R^2 = 1$
ε = 2
$\hat{\alpha}$
α
RMS=0.047
$R^2 = 0.99$
α(1) = 0.05
α(2) = 0.3
π(1) = 0.4
$\hat{\varepsilon}$
ε
RMS(1)=0.024
RMS(2)=0.027
$R^2 = 0.88$
ε = 2
π(1) = 0.4
$\bar{\alpha} = 0.2$
α(1)
α(2)
$\hat{\alpha}$
α
RMS=0.042
$R^2 = 0.97$
ε = 2
α(1) = 0.05
α(2) = 0.3
$\hat{\pi}(1)$
π(1)
