# Supplementary material for: Where Have All the Interactions Gone? Estimating the Coverage of Two-Hybrid Protein Interaction Maps
Source: PLoS Comput Biol. 2007 Nov 23;3(11):e214. doi: 10.1371/journal.pcbi.0030214 (PMC2082503; doi:10.1371/journal.pcbi.0030214)
Supplement: Supplementary file 3 [file pcbi.0030214.sg003.pdf]

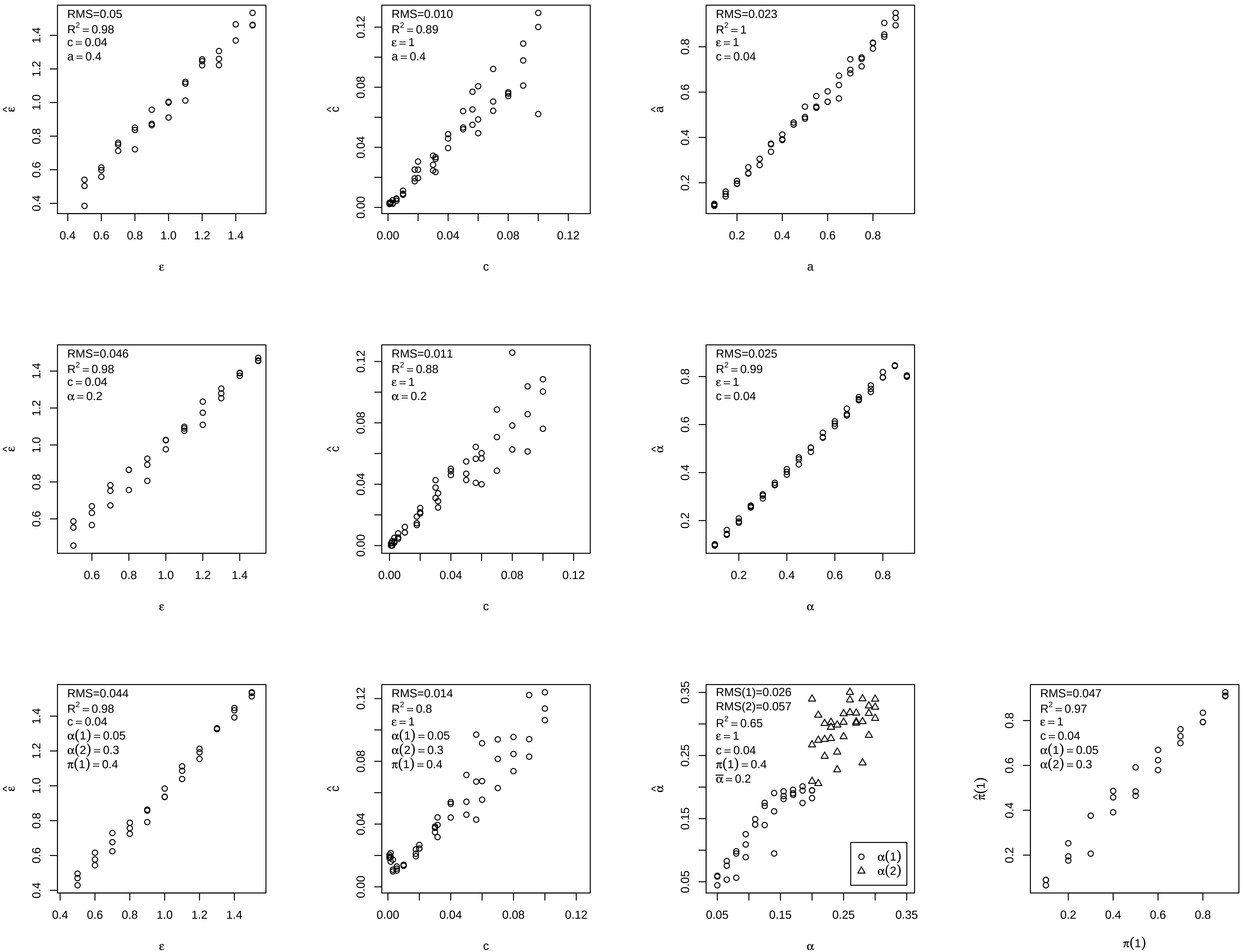

RMS=0.05
R2 = 0.98
c = 0.04
a = 0.4
ε
RMS=0.010
R2 = 0.89
ε = 1
a = 0.4
c
RMS=0.023
R2 = 1
ε = 1
c = 0.04
a
RMS=0.046
R2 = 0.98
c = 0.04
α = 0.2
ε
RMS=0.011
R2 = 0.88
ε = 1
α = 0.2
c
RMS=0.025
R2 = 0.99
ε = 1
c = 0.04
α
RMS=0.044
R2 = 0.98
c = 0.04
α(1) = 0.05
α(2) = 0.3
π(1) = 0.4
ε
RMS=0.014
R2 = 0.8
ε = 1
α(1) = 0.05
α(2) = 0.3
π(1) = 0.4
c
RMS(1)=0.026
RMS(2)=0.057
R2 = 0.65
ε = 1
c = 0.04
π(1) = 0.4
ᾱ = 0.2
α(1)
α(2)
α
RMS=0.047
R2 = 0.97
ε = 1
c = 0.04
α(1) = 0.05
α(2) = 0.3
π(1)
