# Supplementary material for: Where Have All the Interactions Gone? Estimating the Coverage of Two-Hybrid Protein Interaction Maps
Source: PLoS Comput Biol. 2007 Nov 23;3(11):e214. doi: 10.1371/journal.pcbi.0030214 (PMC2082503; doi:10.1371/journal.pcbi.0030214)
Supplement: Supplementary file 4 [file pcbi.0030214.sg004.pdf]

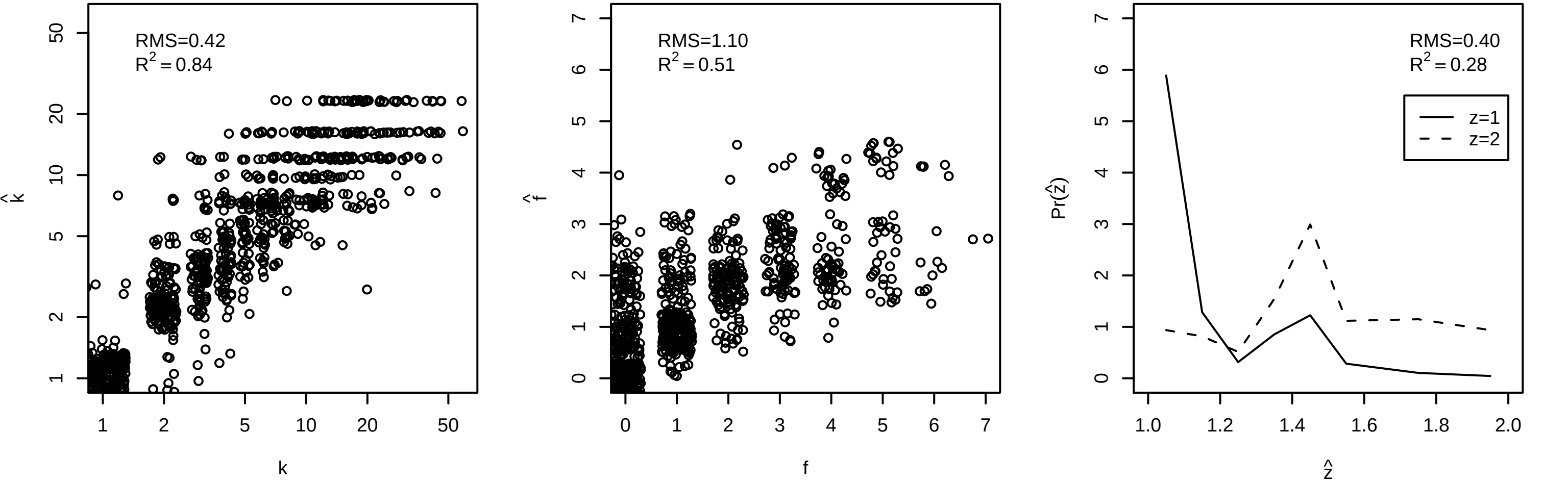

RMS=0.42
$R^2 = 0.84$
k
$\hat{k}$
RMS=1.10
$R^2 = 0.51$
f
$\hat{f}$
RMS=0.40
$R^2 = 0.28$
z=1
z=2
$\hat{z}$
$Pr(\hat{z})$
