# Supplementary material for: Where Have All the Interactions Gone? Estimating the Coverage of Two-Hybrid Protein Interaction Maps
Source: PLoS Comput Biol. 2007 Nov 23;3(11):e214. doi: 10.1371/journal.pcbi.0030214 (PMC2082503; doi:10.1371/journal.pcbi.0030214)
Supplement: Supplementary file 5 [file pcbi.0030214.sg005.pdf]

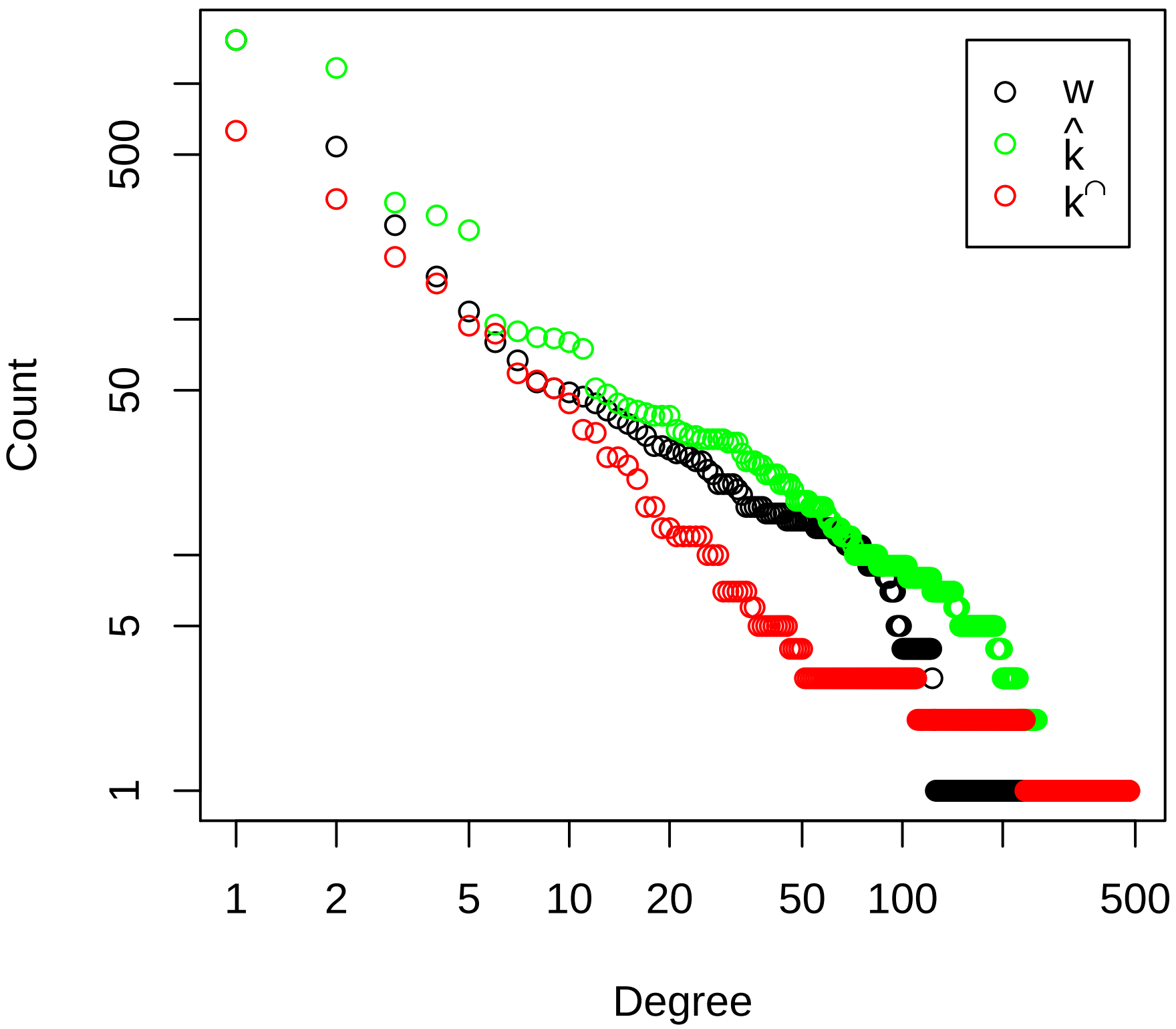

Count
500
50
5
1
Degree
1
2
5
10
20
50
100
500
w
^k
k⌒
