# Supplementary material for: Where Have All the Interactions Gone? Estimating the Coverage of Two-Hybrid Protein Interaction Maps
Source: PLoS Comput Biol. 2007 Nov 23;3(11):e214. doi: 10.1371/journal.pcbi.0030214 (PMC2082503; doi:10.1371/journal.pcbi.0030214)
Supplement: Supplementary file 6 [file pcbi.0030214.st001.doc]

| Deg. dist. | Error Model | Param. | Low | High | RMS |  |
| --- | --- | --- | --- | --- | --- | --- |
| ER | Scaled |  | 3.0 | 5.0 | 0.1 | 0.97 |
|  |  |  | 0.1 | 0.9 | 0.035 | 0.98 |
| ER | Single |  | 3.0 | 5.0 | 0.06 | 1.00 |
|  |  |  | 0.1 | 0.9 | 0.051 | 0.96 |
| ER | Mixture |  | 3.0 | 5.0 | 0.086 | 0.98 |
|  |  |  | 0.05 | 0.2 | 0.034 | 0.73 |
|  |  |  | 0.2 | 0.3 | 0.042 | 0.73 |
|  |  |  | 0.1 | 0.9 | 0.069 | 0.93 |
| PL | Scaled |  | 1.5 | 3.0 | 0.054 | 0.99 |
|  |  |  | 0.1 | 0.9 | 0.018 | 1.00 |
| PL | Single |  | 1.5 | 3.0 | 0.047 | 0.99 |
|  |  |  | 0.1 | 0.9 | 0.0071 | 1.00 |
| PL | Mixture |  | 1.5 | 3.0 | 0.047 | 0.99 |
|  |  |  | 0.05 | 0.2 | 0.024 | 0.88 |
|  |  |  | 0.2 | 0.3 | 0.027 | 0.88 |
|  |  |  | 0.1 | 0.9 | 0.042 | 0.97 |
| TPL | Scaled |  | 0.5 | 1.5 | 0.05 | 0.98 |
|  |  |  | 0.001 | 0.1 | 0.010 | 0.89 |
|  |  |  | 0.1 | 0.9 | 0.023 | 1.00 |
| TPL | Single |  | 1.5 | 3.0 | 0.046 | 0.98 |
|  |  |  | 0.001 | 0.1 | 0.011 | 0.88 |
|  |  |  | 0.1 | 0.9 | 0.025 | 0.99 |
| TPL | Mixture |  | 1.5 | 3.0 | 0.044 | 0.98 |
|  |  |  | 0.001 | 0.1 | 0.014 | 0.80 |
|  |  |  | 0.05 | 0.2 | 0.026 | 0.65 |
|  |  |  | 0.2 | 0.3 | 0.057 | 0.65 |
|  |  |  | 0.1 | 0.9 | 0.047 | 0.97 |
