# Supplementary material for: Where Have All the Interactions Gone? Estimating the Coverage of Two-Hybrid Protein Interaction Maps
Source: PLoS Comput Biol. 2007 Nov 23;3(11):e214. doi: 10.1371/journal.pcbi.0030214 (PMC2082503; doi:10.1371/journal.pcbi.0030214)
Supplement: Supplementary file 7 [file pcbi.0030214.st002.doc]

| Generative | model: | ER | ER | ER | PL | PL | PL | TPL | TPL | TPL |
| --- | --- | --- | --- | --- | --- | --- | --- | --- | --- | --- |
|  |  | SCALED | SINGLE | MIXTURE | SCALED | SINGLE | MIXTURE | SCALED | SINGLE | MIXTURE |
|  | BestBIC | 26654.5 | 26651.5 | 26697.1 | 25516.1 | 22648.1 | 20807.8 | 25653 | 28167.5 | 22754.6 |
| Fitting model, | BIC |  |  |  |  |  |  |  |  |  |
| ER | SCALED | **0** | 135.7 | 232.4 | 1083.2 | 2955.6 | 3051.6 | 971.4 | 1868.5 | 2223 |
| ER | SINGLE | 103.7 | **0** | 42.9 | 772.6 | 1651.7 | 1721.3 | 513.8 | 826.5 | 999.2 |
| ER | MIXTURE | 110.6 | 6.9 | **0** | 258.7 | 334.6 | 360.9 | 272.7 | 202.4 | 362.7 |
| PL | SCALED | 1289.9 | 1294.8 | 1082.7 | **0** | 60.6 | 88.3 | 53.4 | 490.5 | 174.1 |
| PL | SINGLE | 1784.4 | 1529.1 | 1330.2 | 290.9 | **0** | 2.6 | 291.3 | 425.7 | 128.4 |
| PL | MIXTURE | 1784.8 | 1532.9 | 1312.4 | 271.9 | 42.2 | **0** | 284.4 | 426 | 130.7 |
| TPL | SCALED | 0.1 | 83.7 | 78.3 | 7.7 | 70.2 | 88.7 | **0** | 51.7 | 49.5 |
| TPL | SINGLE | 39 | 20.2 | 30.8 | 288.7 | 1.4 | 1.9 | 174.6 | **0** | 3.2 |
| TPL | MIXTURE | 45.9 | 27.1 | 7.9 | 267.8 | 12.7 | 18 | 166.9 | 5.3 | **0** |
