# Supplementary material for: Where Have All the Interactions Gone? Estimating the Coverage of Two-Hybrid Protein Interaction Maps
Source: PLoS Comput Biol. 2007 Nov 23;3(11):e214. doi: 10.1371/journal.pcbi.0030214 (PMC2082503; doi:10.1371/journal.pcbi.0030214)
Supplement: Supplementary file 8 [file pcbi.0030214.st006.doc]

|  | **Yeast** |  |  |  | **Worm** |  |  |  | **Fly** |  |  |  |
| --- | --- | --- | --- | --- | --- | --- | --- | --- | --- | --- | --- | --- |
|  | **# of baits** | **Fraction of baits** |  |  | **# of baits** | **Fraction of baits** |  |  | **# of baits** | **Fraction of baits** |  |  |
| **1** | 717 | 46.8% | 2.52 | 0.07 | 190 | 26.1% | 3.92 | 0.12 | 664 | 18.2% | 3.43 | 0.14 |
| **2** | 135 | 8.8% | 5.79 | 0.32 | 69 | 9.5% | 6.28 | 0.36 | 165 | 4.5% | 5.14 | 0.43 |
| **3** | 24 | 1.6% | 11.89 | 0.45 | 26 | 3.6% | 9.23 | 0.56 | 44 | 1.2% | 7.02 | 0.72 |
| **4** | 5 | 0.3% | 20.77 | 0.55 | 12 | 1.6% | 12.43 | 0.73 | 28 | 0.8% | 8.96 | 1.01 |
| **5** | 3 | 0.2% | 32.51 | 0.63 | 6 | 0.8% | 15.69 | 0.9 | 22 | 0.6% | 10.94 | 1.31 |
| **6** | 1 | 0.1% | 47.17 | 0.70 | 2 | 0.3% | 18.95 | 1.08 | 9 | 0.2% | 12.93 | 1.63 |
| **7** | 0 | 0 | NA | NA | 4 | 0.5% | 22.18 | 1.25 | 7 | 0.2% | 14.89 | 1.95 |
| **8** | 0 | 0 | NA | NA | 0 | 0 | NA | NA | 8 | 0.2% | 16.83 | 2.29 |
| **9** | 0 | 0 | NA | NA | 1 | 0.1% | 28.59 | 1.61 | 3 | 0.1% | 18.72 | 2.65 |
| **10** | 0 | 0 | NA | NA | 1 | 0.1% | 31.77 | 1.8 | 1 | 0.03% | 20.57 | 3.02 |
| **11** | 0 | 0 | NA | NA | 1 | 0.1% | 34.93 | 1.98 | 3 | 0.1% | 22.39 | 3.4 |
| **12** | 1 | 0.1% | 190.13 | 1.16 | 0 | 0 | NA | NA | 3 | 0.1% | 24.17 | 3.8 |
| **13** | 1 | 0.1% | 222.73 | 1.24 | 0 | 0 | NA | NA | 0 | 0 | NA | NA |
| **14** | 0 | 0 | NA | NA | 0 | 0 | NA | NA | 1 | 0.03% | 27.65 | 4.62 |
| **22** | 0 | 0 | NA | NA | 0 | 0 | NA | NA | 1 | 0.03% | 40.97 | 8.16 |
| **23** | 0 | 0 | NA | NA | 0 | 0 | NA | NA | 1 | 0.03% | 42.62 | 8.61 |
| **35** | 0 | 0 | NA | NA | 1 | 0.1% | 108.21 | 7.12 | 0 | 0 | NA | NA |
